# ASIC1a regulates insular long-term depression and is required for the extinction of conditioned taste aversion

**DOI:** 10.1038/ncomms13770

**Published:** 2016-12-07

**Authors:** Wei-Guang Li, Ming-Gang Liu, Shining Deng, Yan-Mei Liu, Lin Shang, Jing Ding, Tsan-Ting Hsu, Qin Jiang, Ying Li, Fei Li, Michael Xi Zhu, Tian-Le Xu

**Affiliations:** 1 Discipline of Neuroscience, Department of Anatomy, Histology and Embryology, and Collaborative Innovation Center for Brain Science, Shanghai Jiao Tong University School of Medicine, Shanghai 200025, China; 2 Department of Developmental and Behavioral Pediatrics, Shanghai Institute of Pediatric Translational Medicine, Shanghai Children's Medical Center, Ministry of Education–Shanghai Key Laboratory of Children's Environmental Health, Shanghai Jiao Tong University School of Medicine, Shanghai 200129, China; 3 Institute of Neuroscience, National Yang-Ming University, 155, Section 2, Li-Nong Street, Taipei 112, Taiwan; 4 Department of Integrative Biology and Pharmacology, The University of Texas Health Science Center at Houston, Houston, Texas 77030, USA

## Abstract

Acid-sensing ion channel 1a (ASIC1a) has been shown to play important roles in synaptic plasticity, learning and memory. Here we identify a crucial role for ASIC1a in long-term depression (LTD) at mouse insular synapses. Genetic ablation and pharmacological inhibition of ASIC1a reduced the induction probability of LTD without affecting that of long-term potentiation in the insular cortex. The disruption of ASIC1a also attenuated the extinction of established taste aversion memory without altering the initial associative taste learning or its long-term retention. Extinction of taste aversive memory led to the reduced insular synaptic efficacy, which precluded further LTD induction. The impaired LTD and extinction learning in ASIC1a null mice were restored by virus-mediated expression of wild-type ASIC1a, but not its ion-impermeable mutant, in the insular cortices. Our data demonstrate the involvement of an ASIC1a-mediated insular synaptic depression mechanism in extinction learning, which raises the possibility of targeting ASIC1a to manage adaptive behaviours.

## Abstract (web summary)

The acid-sensing ion channel, ASIC1a, is known to play a role in synaptic transmission and plasticity. Here, the authors demonstrate a role for ASIC1a in regulating plasticity in the insular cortex and find that extinction of conditioned taste aversion memory is disrupted in the ASIC1a knockout mice.

Acid-sensing ion channel (ASIC) family comprises six isoforms (ASIC1a, 1b, 2a, 2b, 3 and 4) encoded by four genes^1^,^2^. These proton-gated channels are formed by three identical or different subunits^2^,^3^, with varying pH sensitivity, ion selectivity and pharmacological characteristics depending on subunit compositions. ASIC1a-containing channels are the major ASICs in the central nervous system^4^,^5^, where ASIC1a null neurons exhibit no acid-evoked current^6^. The importance of ASIC1a has been well documented in rodent models of ischaemic neuronal death^7^,^8^, chronic pain^9^,^10^,^11^, seizure termination^12^ and neurodegenerative diseases^13^. ASIC1a is also implicated in affective disorders, including anxiety^14^ and depression^15^. However, despite the rich information about its neuronal expression and contributions to pathological processes, the physiological role(s) of ASIC1a in brain neurons remains largely unknown.

Increasing evidence supports the critical role of ASIC1a in synaptic transmission and plasticity. Given its postsynaptic localization^6^,^16^, ASIC1a has been postulated to be activated by synaptically released protons during normal neurotransmission^17^,^18^. ASIC1a serves as the main synaptic proton receptor to regulate high-frequency stimulation or theta burst stimulation (TBS)-induced long-term potentiation (LTP) in hippocampus^6^,^19^,^20^ and amygdala^17^,^21^, and contribute to fear learning^14^,^16^,^21^. ASIC1a promotes spine density in hippocampus^22^, but inhibits spine density, alters the excitatory synaptic receptor function and constrains cocaine-evoked plasticity in nucleus accumbens^18^, suggesting complex roles of ASIC1a in modulating synaptic plasticity and behavioural adaptation.

The complexity of ASIC1a function may reflect region-specific participation in different forms of synaptic plasticity and behavioural regulation. ASIC1a is highly expressed in neurons in the insular cortex^1^,^16^, but its function there is unclear. Insular cortex is critical for cognition and emotion control^23^, including sensory integration^24^, chronic pain^25^,^26^, emotional processing^24^,^27^ and gustatory recognition memory^28^. It is best known for its role in taste learning^29^ and processing aversively motivated learning tasks, that is, conditioned taste aversion (CTA)^30^,^31^,^32^,^33^, a form of associative learning where the subject associates a novel taste (conditioned stimulus) with a subsequent transient visceral illness (unconditioned stimulus, US). At the synaptic level, LTP in insular cortex is known to contribute to the acquisition of CTA^34^,^35^. However, the molecular and synaptic mechanisms underlying CTA memory extinction^36^,^37^ remain undetermined. Here we examine the role of ASIC1a in synaptic plasticity in the adult mouse insular cortex using multiple-electrode array slice recording^38^,^39^, and CTA learning and extinction by behavioural assessment. We found that in contrast to the established role for ASIC1a in LTP, which promotes associative learning and memory in other brain areas^6^,^16^,^21^, ASIC1a is a critical modulator of long-term depression (LTD) in the insular cortex and this function is important for the extinction of the acquired taste aversion memory.

## Results

### Prominent ASIC1a expression in mouse insular cortex

We first verified ASIC1a expression in insular cortices of adult mice^1^,^16^. ASIC1a messenger RNA was the most abundant subtype in insular cortical preparations from wild-type (WT) mice, but absent in that from *ASIC1a* knockout (KO) mice (Supplementary Fig. 1a,b). ASIC1a protein was also abundant in insular cortices of WT mice, comparable with that in prefrontal cortex and amygdala, richer than that in hippocampus, and was completely absent in *ASIC1a* KO animals (Supplementary Figs 1c and 13d). These data demonstrate a prominent ASIC1a expression in insular cortex.

### Normal insular glutamatergic transmission in ASIC1a KO

To examine the basal synaptic role of ASIC1a in insular cortex, we compared several major components of postsynaptic density (PSD) between insular cortices from WT and *ASIC1a* KO mice. We found no significant difference in synaptic NMDA receptor (NMDAR) subunits GluN1, GluN2A, GluN2B, as well as its principal scaffold protein, PSD protein 95 (PSD-95) and AMPA receptor (AMPAR) subunits GluA1 and GluA2 (Supplementary Figs 1d,e and 13e). Electrophysiological recordings of pyramidal neurons^25^,^26^ in layer II and III of acutely isolated insular cortical slices revealed no difference between WT and *ASIC1a* KO samples in intrinsic excitability (Supplementary Fig. 2a–d), input–output curves of excitatory postsynaptic currents (EPSCs; Supplementary Fig. 2e), paired-pulse ratios (Supplementary Fig. 2f), miniature EPSCs (Supplementary Fig. 2g–i) and AMPAR/NMDAR ratios (Supplementary Fig. 2j,k), suggesting comparable basal synaptic transmission.

### ASIC1a is required for low-frequency stimulation-induced LTD

**Figure 1:**
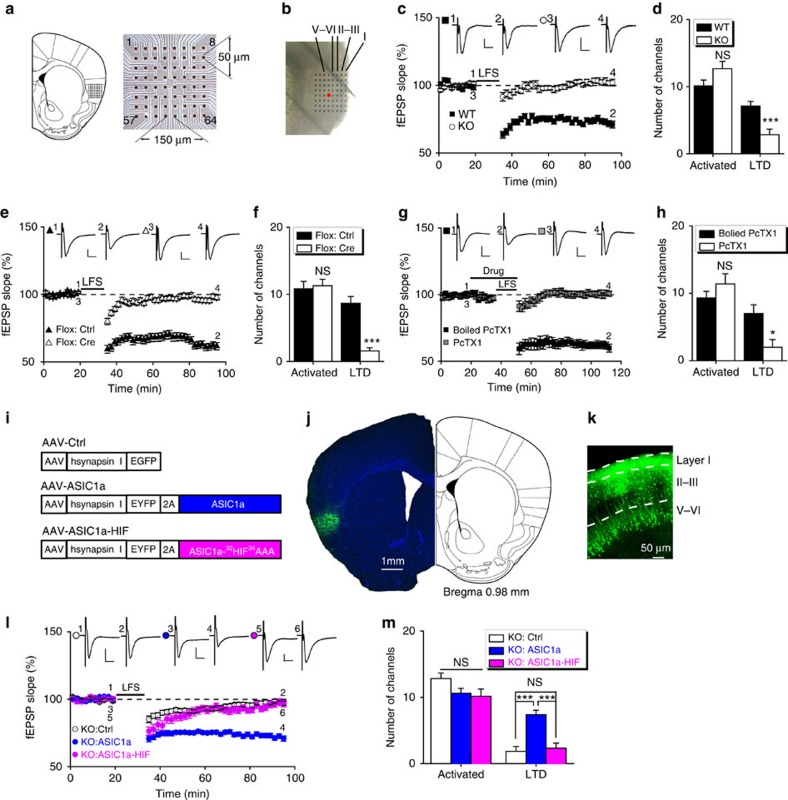
ASIC1a is important for LFS-evoked LTD in insular cortex. (**a**) Schematic diagram showing the location of a MED64 probe placed on the coronal insular slice (left) and the arrangement of the 8 × 8 recording array. (**b**) Light microscopy photograph showing the location of the MED64 probe relative to the insular cortex and the layer (from I to V and VI) designation. The red dot indicates the stimulation site. (**c**,**e**,**g**,**l**) Time courses of fEPSP slope changes in response to LFS (1 Hz, 900 pulses) at insular slices under conditions indicated. Inset: representative fEPSP traces at the time points indicated by numbers in the graph. Scale bar, 100 μV, 10 ms. (**d**,**f**,**h**,**m**) Summary of number of activated channels per slice and those that showed LTD in insular synapses. NS, not significant, *P<0.05, ***P<0.001, (**d**) *P*=0.1570 and 0.0005, *n*=7–9 slices per 7–8 mice; (**f**) *P*=0.7632 and 3.854E−05, *n*=6–7 slices per 4 mice each; (**h**) *P*=0.2660 and 0.0194, *n*=5–6 slices per 3–5 mice, compared between two groups; (**m**) activated: *P*=0.0700, 0.0787 and 0.7211, LTD: *P*=0.0002, 0.6495 and 0.0004, *n*=6–8 slices per 4–8 mice, compared with Ctrl versus ASIC1a, Ctrl versus ASIC1a-HIF and ASIC1a versus ASIC1a-HIF, respectively, unpaired Student's *t*-test. (**i**) Schematics of AAV vectors engineered to express a control construct (EGFP), ASIC1a or a pore-dead mutant (^32^HIF^34^AAA) of ASIC1a. Hsynapsin I, the human synapsin I promoter that drives expression in neurons. (**j**) An example of AAV-mediated EYFP expression in the insular cortex (left). The slice was counterstained with DAPI (4',6-diamidino-2-phenylindole). A brain atlas at the similar brain section level that contains the insular cortex (Bregma +0.98 mm) is shown at right. (**k**) A representative image at high magnification showing EYFP expression in individual insular neurons.

We then examined a variety of synaptic plasticity models using a previously established 64-channel multi-electrode array (MED64) recording system^38^,^39^, with the MED64 probe placed within the insular cortical slice as shown in Fig. 1a,b. We recorded the responses of the rostral insular region at the level of the corpus callosum connection to stimulation in the deep layers (V and VI) of the insular slice (the red dot in Fig. 1b). Current injection at the stimulation site typically elicited field excitatory postsynaptic potentials (fEPSPs) in a fraction of channels (∼10 channels) of the 64-channel array. These are referred to as activated channels. Consistent with the previous study^39^, low-frequency stimulation (LFS) in insular cortical slices from WT mice induced a significant depression of fEPSPs in the majority of activated channels (Fig. 1c; Supplementary Fig. 3a–c), hence designated as LFS–LTD. This was abolished by NMDAR antagonist D-AP5 and therefore also referred to as NMDAR-dependent LTD (NMDAR-LTD)^39^. The induction ratio, defined as the percentage of LTD-positive channels among all activated channels, was 70.5±4.9% (Fig. 1d; Supplementary Fig. 3d,e) in WT, but significantly decreased to 20.9±5.7% in *ASIC1a* KO mice (*P*=1.156E−05 versus WT; Fig. 1d; Supplementary Fig. 3f–j).

For channels that displayed LTD, the loss of ASIC1a did not appear to affect its magnitude, as the mean LTD magnitude of all LTD-positive channels were not significantly different between WT and *ASIC1a* KO groups (Supplementary Fig. 4a). The fEPSP amplitudes of activated channels did not influence the probability of LTD induction. When only channels with the strongest responses from individual slices were used for quantification, the mean fEPSP slopes at 50–60 min following LFS were significantly decreased in WT, but not *ASIC1a* KO groups (Supplementary Fig. 4b). Furthermore, neurons in both superficial (layer II–III) and deep (layer V–VI) layers of insular cortex exhibited reduced LTD induction (LTD induction ratios: superficial, WT: 73.7±7.4%, *ASIC1a* KO: 24.9±11.0%, *P*=0.0019; deep: WT: 69.9±6.1%, *ASIC1a* KO: 18.9±8.5%, *P*=0.0002; Supplementary Figs 4c,d and 8a,b) in *ASIC1a* KO mice. Together, these data suggest a global role of ASIC1a in the probability of insular LTD induction, but not LTD magnitudes in individual neurons, and this effect is independent of the fEPSP amplitude and cortical layer specificity of insular neurons.

To further establish the role of ASIC1a in insular LTD, we used a conditional *ASIC1a* KO line (*ASIC1a*^*flox/flox*^)^19^, to which an adeno-associated virus (AAV) that contained the coding sequence for either green fluorescence protein (GFP, AAV-Ctrl) or Cre recombinase (AAV-Cre) was injected bilaterally into insular cortices ∼1 month before slice recording by the MED64 system. Western blotting showed that the injection of AAV-Cre reduced ASIC1a protein expression in insular cortex as compared with AAV-Ctrl (Supplementary Figs 5a,b and 13f). The LTD induction ratio reduced from 80.0±4.3% for AAV-Ctrl- to 15.9±5.5% for AAV-Cre-injected *ASIC1a*^*flox/flox*^ mice (*P*=2.177E−06 versus AAV-Ctrl; Fig. 1e,f).

We next examined the effect of pharmacological blockade of ASIC1a-containing channels on LFS–LTD in insular cortex. Psalmotoxin 1 (PcTX1, 100 nM), an inhibitor of homomeric ASIC1a and heteromeric ASIC1a/2b channels^40^,^41^, was applied to the slice at ∼15 min before and continued during the LFS delivery. Heat-inactivated (boiled) PcTX1 was used as a control. Neither PcTX1 nor boiled PcTX1 altered basal excitatory synaptic transmission (Fig. 1g); however, the insular LTD induction ratio decreased from 72.4±8.4% for boiled PcTX1 to 17.9±9.3% for PcTX1-tretated slices (*P*=0.0018; Fig. 1h). These results confirm the critical role of ASIC1a in insular LTD and rule out the possibility that LTD deficit in the *ASIC1a* KO mice might be caused by developmental compensation.

### Re-expression of ASIC1a in ASIC1a null mice rescues LFS–LTD

To test whether restoration of ASIC1a expression in the insular cortices of *ASIC1a* KO mice could rescue LTD induction, we prepared an AAV that contained the coding sequence for enhanced yellow fluorescence protein (EYFP) protein linked to the N terminus of mouse ASIC1a through a ‘self-cleaving' 2A-peptide^42^ driven by the human synapsin I promoter (AAV-ASIC1a; Fig. 1i) for neuron-specific expression. AAV-ASIC1a and the control virus (AAV-Ctrl; Fig. 1i) were bilaterally injected into the insular cortices of *ASIC1a* KO mice (Fig. 1j,k) ∼1 month before slice recording. The injection of AAV-ASIC1a, but not AAV-Ctrl, successfully rescued LFS–LTD (Fig. 1l), with the induction ratio increased from 14.9±6.3% for AAV-Ctrl to 69.9±5.4% for AAV-ASIC1a (*P*=2.513E-05 versus AAV-Ctrl; Fig. 1m). Moreover, expression of a non-conducting mutant, ASIC1a-HIF (^32^HIF^34^ mutated to ^32^AAA^34^; Fig. 1i), which has been shown to be devoid of channel conductance due to pore dysfunction^43^, failed to rescue the impaired insular LTD induction in *ASIC1a* KO mice (Fig. 1l), with the induction ratio (24.0±8.2%, *P*=0.3976; Fig. 1m) not significantly different from that of AAV-Ctrl. These data, thus, support the critical contribution of ASIC1a and its ion conductance to insular LTD and again rule out developmental compensation, as a contributing factor of impaired LTD in the mutant mice.

### Importance of pH dynamics for LFS–LTD in insular cortex

As proton-gated channels, ASIC1a most likely contributes to LFS–LTD in response to pH fluctuations during synaptic activities. To test the influence of pH dynamics on LTD induction, we increased the buffering capacity of extracellular solution during LFS according to a previous protocol shown to reduce ASIC activation in neurons^18^. As expected, this treatment greatly reduced the probability of LTD induction in insular cortices of WT mice (Supplementary Fig. 6), supporting the idea that ASIC1a acts in response to changes in extracellular pH to modulate LTD induction in insular synapses.

### ASIC1a is critical for DHPG-induced LTD in insular cortex

**Figure 2:**
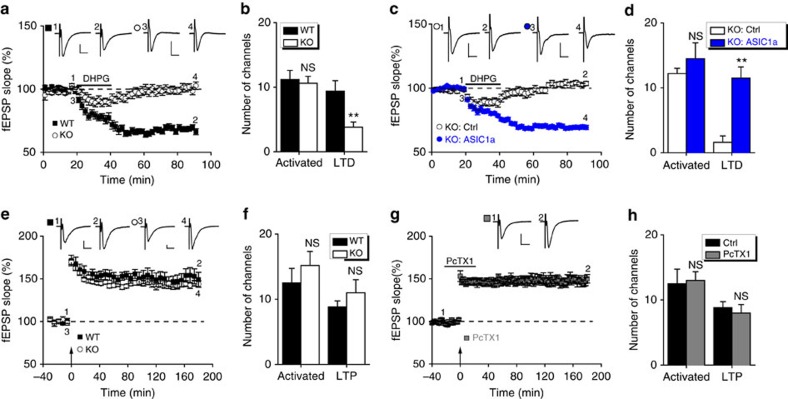
ASIC1a is important for DHPG-induced LTD but not TBS-induced LTP in insular cortex. (**a**,**c**,**e**,**g**) Time courses of fEPSP slope changes in response to application of DHPG (100 μM, 20 min, **a**,**c**) and TBS (10 bursts at 5 Hz, 4 pulses at 100 Hz for each burst, **e**,**g**) to insular slices under conditions indicated ((**a**,**e**) WT versus ASIC1a KO; (**c**) ASIC1a KO, infected with control (Ctrl) or ASIC1a-WT AAV; (**g**) WT, treated with PcTX1 (100 nM, initiated at 20 min before the TBS protocol and sustained for a total duration of 40 min as shown)). Inset: representative fEPSP traces at the time points indicated by numbers in the graph. Scale bar, 100 μV, 10 ms. (**b**,**d**,**f**,**h**) Summary of number of activated channels per slice and those that showed DHPG-LTD (**b**,**d**) or TBS-LTP (**f**,**h**) at insular synapses. NS, not significant, **P<0.01, (**b**) *P*=0.7420 and 0.0028, *n*=5 slices per 4 mice each; (**d**) *P*=0.4301 and 0.0011, *n*=5–6 slices per 5–6 mice; (**f**) *P*=0.4083 and 0.3441, *n*=6 slices per 6 mice each; (**h**) *P*=0.8597 and 0.6035, *n*=5–6 slices per 5–6 mice, compared between two groups, unpaired Student's *t*-test. The Ctrl data in **h** were taken from **f** and regraphed for comparison.

We further looked into the role of ASIC1a in another form of synaptic depression induced by bath application of group I metabotropic glutamate receptor agonist, 3,5-dihydroxyphenylglycine (DHPG, 100 μM), which is resistant to inhibition by NMDAR antagonist and thus referred to as NMDAR-independent LTD^39^. Notably, DHPG-induced LTD (DHPG-LTD) was also disrupted in the mutant animals (Fig. 2a). The induction ratio of insular DHPG-LTD was decreased from 82.2±5.6% for WT littermates to 20.4±9.2% for *ASIC1a* KO mice (*P*=0.0004; Fig. 2b). Importantly, the expression of AAV-ASIC1a, but not AAV-Ctrl, in insular cortices of *ASIC1a* KO mice restored DHPG-LTD induction ratio to the WT level (AAV-ASIC1a, 82.2±5.8%; AAV-Ctrl, 15.3±9.4%, *P*=0.0001; Fig. 2c,d). Thus, ASIC1a is important for the development of both NMDAR-dependent and NMDAR-independent LTD in mouse insular cortex.

### ASIC1a is not required for LTP induction in insular cortex

We also examined the impact of ASIC1a deletion/inhibition on LTP, another form of synaptic plasticity in insular cortex. Consistent with the previous study^38^, TBS in WT insular cortical slices induced a rapid and long-lasting potentiation of synaptic response in multiple sites (Fig. 2e; Supplementary Fig. 7a–c). However, the TBS-induced LTP reached similar levels between slices prepared from WT and *ASIC1a* KO mice (Fig. 2e; Supplementary Fig. 7d–f), with LTP induction ratios averaged to 75.5±7.4% for WT and 71.2±6.3% for *ASIC1a* KO samples (*P*=0.6711; Fig. 2f), and no cortical layer specificity (Supplementary Fig. 8c,d). Moreover, the ASIC1a inhibitor, PcTX1, did not affect TBS induction of LTP in insular cortical slices from WT mice (Fig. 2g), with an LTP induction ratio of 60.9±6.6% (Fig. 2h), comparable to that of untreated WT samples (*P*=0.1822). These results indicate that ASIC1a is unlikely involved in insular LTP induction by TBS, which contrasts sharply to its pronounced contribution to insular LTD induction as described above.

### Insular LTD is selectively abolished by CTA extinction

In light of the selective role of ASIC1a in insular LTD, we sought to determine the specific insular cortex-regulated behavioural responses to which LTD might have a critical contribution. As stated in the Introduction, insular cortex is important for CTA, a form of aversively motivated taste learning task^30^,^31^,^32^,^33^. While insular LTP has been linked to CTA acquisition^34^,^35^, little is known about the role of insular LTD in this process. Given the usually opposing roles of LTP and LTD in synaptic plasticity, we suspected that insular LTD might play a role in CTA extinction. As such, insular cortical slices from mice subjected to CTA training, including acquisition, retention and extinction, might exhibit altered LTD induction, depending on the specific process, in which insular LTD executes its function.

**Figure 3:**
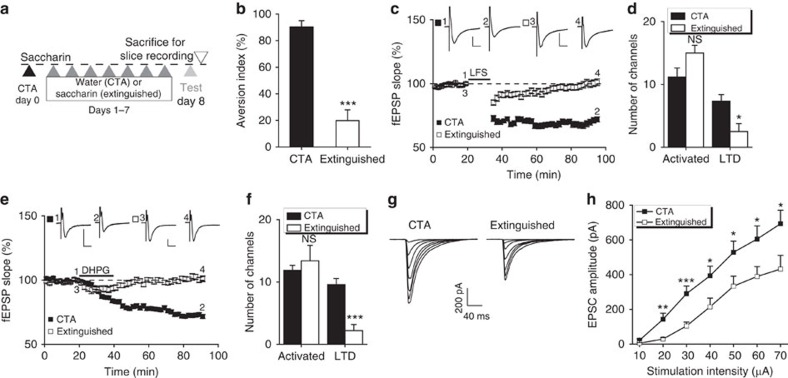
Insular LTD is involved in CTA memory extinction. (**a**) Schematic representation of the behavioural protocols to establish (association of saccharin solution with LiCl injection on day 0) and retain (by giving water only on days 1–7) or extinguish (by giving saccharin solution only on days 1–7) CTA before slices were prepared for studying LTD induction *ex vivo*. (**b**) The aversion index of mice (tested by the choices between two bottles: water and saccharin solution) subjected to forced CTA extinction (extinguished) or CTA retention (CTA) for seven consecutive days. *n*=5–7 per group. ***P<0.001, *P*=1.109E−05, unpaired Student's *t*-test. (**c**–**f**) LTD induction in insular cortex was abolished in CTA memory extinguished, but not CTA retained (CTA) mice. (**c**,**e**) Time courses of fEPSP slope changes in response to LFS (**c**) or DHPG (**e**) in neurons of insular slices from CTA-extinguished and retained mice. Inset: representative fEPSP traces at the time points indicated by numbers in the graph. Scale bar, 100 μV, 10 ms. (**d**,**f**) Summary of number of activated channels per slice and those that exhibited LTD. NS, not significant, *P<0.05, ***P<0.001, (**d**) *P*=0.0719 and 0.0147, *n*=6 slices per 4 mice each; (**f**) *P*=0.2915 and 0.0001, *n*=5–7 slices per 4 mice each, CTA versus extinguished, unpaired Student's *t*-test. (**g**,**h**) Input–output relationships of insular cortical neurons of CTA-extinguished and retained mice. (**g**) Representative traces. (**h**) Summary data. *n*=20–23 cells per 7 mice each. *P<0.05, **P<0.01, ***P<0.001, *P*=0.0024, 0.0004, 0.0217, 0.0295, 0.0288 and 0.0236, CTA versus extinguished, unpaired Student's *t*-test.

To induce CTA, mice were presented in the drinking water with saccharin as conditioned stimulus, which was then followed by the intraperitoneal injection of LiCl to cause malaise (US). The establishment of CTA was confirmed using aversion index, which compares the consumption of water and saccharin solution in two drinking bottles presented to the conditioned animal during the 30-min drinking period in subsequent days (Supplementary Fig. 9a). With this protocol, most animals acquired aversion index values between 80–90% after conditioning (see below). To extinguish CTA, the animals were allowed to drink only the saccharin solution (extinguished) during the 30-min drinking period for 7 days, whereas the control animals were given water only (CTA) to maintain the taste aversion (Fig. 3a). As shown in Fig. 3b, the CTA control mice maintained the high aversion index, while the extinguished animals exhibited a markedly reduced aversion index, indicating the extinction of CTA by the consumption of saccharin without the association with LiCl injection.

Analysing insular cortical slices from the above mice, we found that the CTA controls without extinction exhibited normal LFS–LTD (Fig. 3c), with an induction ratio of 68.5±7.5% (Fig. 3d). For DHPG-induced LTD, the induction ratio was 80.3±2.6% (Fig. 3f). Both of these values were comparable with those from naive mice (Figs 1d and 2b), indicating that insular LTD is not involved in the acquisition and retention of CTA. On the other hand, slices from the CTA-extinguished mice displayed diminished LTD to LFS (Fig. 3c) or DHPG (Fig. 3e). The LTD induction ratios were 19.3±11.4% (Fig. 3d; *P*=0.0049) and 11.4±5.5% (Fig. 3f; *P*=1.982E-07) in response to LFS and DHPG, respectively, which were significantly different from that of CTA controls. These results demonstrate that CTA extinction but not its acquisition or retention abolishes insular LTD.

The loss of LTD could result from blockade of mechanism(s) involved in LTD induction or a saturation effect that occludes subsequent LTD. To distinguish between these possibilities, we compared synaptic efficacy in insular cortical slices from CTA control and extinguished mice. We reasoned that if the LTD induction mechanism was disrupted, the basal excitatory synaptic efficacy should not differ between the two groups, whereas if LTD was executed during CTA extinction, the synaptic efficacy should be decreased. The input–output curves obtained by plotting the EPSC amplitude against stimulating intensity revealed a significant reduction of the synaptic efficacy in CTA extinguished compared with control samples (Fig. 3g,h), suggesting that CTA extinction indeed depressed excitatory synaptic transmission in insular cortex. Therefore, insular LTD is likely involved in the extinction of CTA memory. This resulted in a saturation effect, which occluded LTD induction in subsequent analyses using insular cortical slices from CTA-extinguished animals.

### Loss of ASIC1a selectively impairs CTA extinction

**Figure 4:**
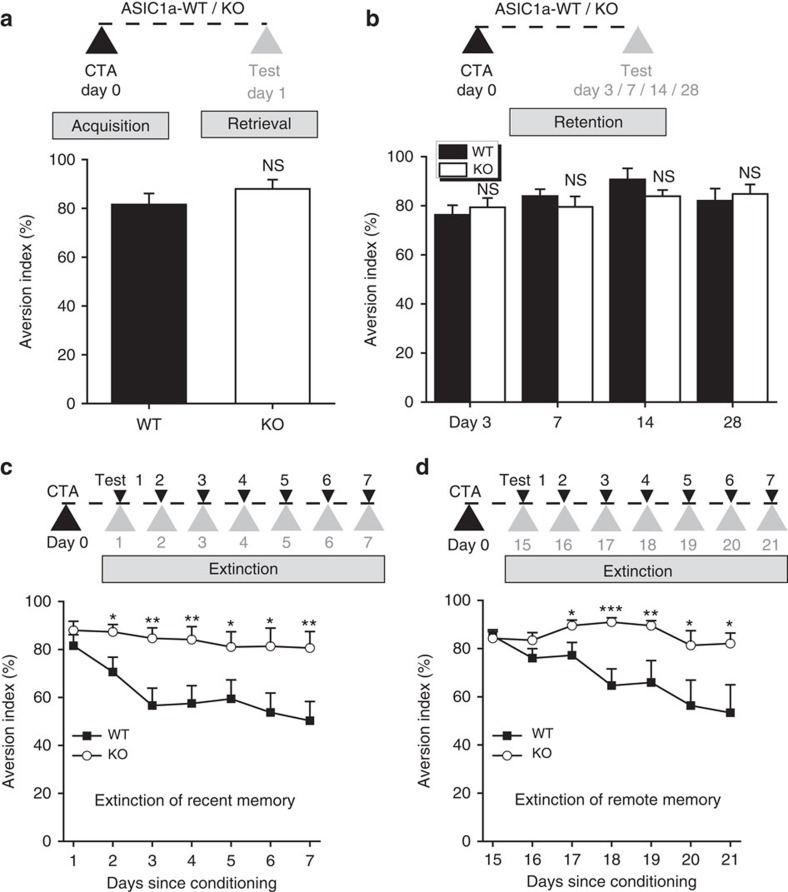
ASIC1a is important for extinction but not acquisition or retention of CTA memory. WT and ASIC1a KO mice were subjected to CTA on day 0. The acquisition of CTA memory was tested on the second day (day 1, **a**) and the retention examined on days 3, 7, 14 and 28 (**b**) (protocols shown in the upper panels) by the two-bottle choice test and quantified as aversion index (bottom panels). For retention test, the animals were given only water after the conditioning day until the test day. For extinction test (**c**,**d**), animals were given water and the saccharin solution for 30 min on each test day and aversion indices were obtained for seven consecutive days beginning either day 1 after CTA acquisition (for extinction of recent memory, **c**) or day 15 after CTA acquisition and two bottles of water provided on days 1–14 (for extinction of remote memory, **d**). Data for day 1 in **c** were the same as in **a**, regraphed again for comparison. *n*=8–16, NS, not significant, *P<0.05, **P<0.01, ***P<0.001, *P*=0.2916 (**a**); 0.7197, 0.3763, 0.1644 and 0.6585 (**b**); 0.0256, 0.0031, 0.0080, 0.0441, 0.0200 and 0.0082 (**c**); 0.0308, 0.0005, 0.0096, 0.0417 and 0.0186 (**d**), WT versus ASIC1a-KO, unpaired Student's *t*-test. (**c**) Group, F_(1,203)_=42.041, *P*<0.001; test day, F_(6,203)_=2.207, *P*=0.044; interaction, F_(6,203)_=0.847, *P*=0.535; (**d**) group, F_(1,161)_=34.259, *P*<0.001; test day, F_(6,161)_=2.795, *P*=0.013; interaction, F_(6,161)_=2.049, *P*=0.063, two-way analysis of variance.

Given the importance of ASIC1a in insular LTD induction, the ASIC1a null mice would be expected to exhibit normal CTA acquisition and retention, but a dysfunction in CTA extinction. Indeed, CTA acquisition was comparable between WT and *ASIC1a* KO mice. On the day of conditioning, the two groups of mice consumed similar amounts of saccharin solution (saccharin solution intake: WT, 1.93±0.11 g, *n*=15; *ASIC1a* KO: 1.74±0.15 g; *n*=14; *P*=0.3020). On the first day after conditioning, both groups developed aversion to saccharin (Fig. 4a), implying that ASIC1a does not play a significant role in CTA acquisition and retrieval. Over the next 28 days, the retention of CTA memory was determined on days 3, 7, 14 and 28, and shown to be similar between WT and *ASIC1a* KO mice (Fig. 4b), indicating that just like their WT littermates, the ASIC1a null mice were able to retain the normal CTA memory for at least 1 month.

By contrast, the *ASIC1a* KO mice failed to show a reduction in recent CTA memory following repeated choice test in the absence of LiCl, whereas WT mice exhibited CTA extinction within the second to third days of the choice test (Fig. 4c). Similarly, extinction of remote CTA memory, examined on days 15–21 after the conditioning, was also disrupted in the *ASIC1a* KO mice (Fig. 4d). These results indicate that ASIC1a selectively contributes to extinction, but not acquisition or retention of CTA.

As a control, we confirmed that the ASIC1a null mice had intact taste discrimination. Using the two-bottle unconditioned taste preference test^44^, we examined their preference for four taste solutions (sweet, salty, bitter and acidic) over water and found no difference between WT and *ASIC1a* KO littermates (Supplementary Fig. 9b–e).

### ASIC1a in insular cortex is critical for CTA extinction

**Figure 5:**
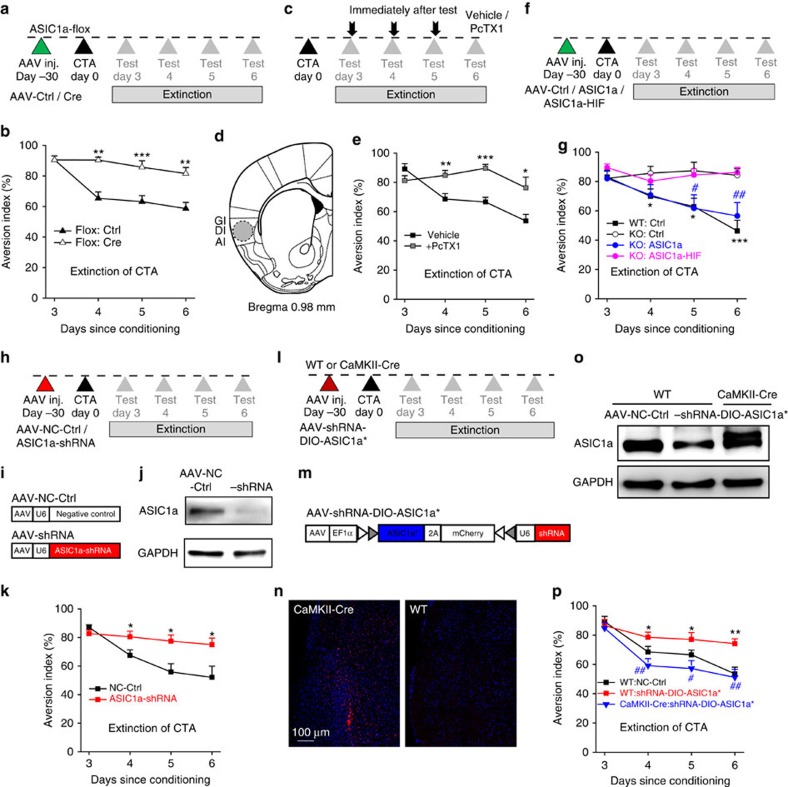
ASIC1a in insular cortex is functionally critical for CTA extinction. (**a**,**c**,**f**,**h**,**l**) Behavioural protocols. (**b**,**e**,**g**,**k**,**p**) Time courses of aversion indices over a 4-day period for animals subjected to the CTA extinction protocol. (**b**) *n*=7. **P<0.01, ***P<0.001, *P*=0.0050, 4.399E−05 and 0.0015, AAV-Ctrl versus AAV-Cre. (**e**) *n*=10–11. *P<0.05, **P<0.01, ***P<0.001, *P*=0.0049, 2.7261E−05 and 0.0136, vehicle versus PcTX1. (**g**) *n*=8–13. *P<0.05, ***P<0.001, *P*=0.0390, 0.0104 and 0.0002, WT+AAV-Ctrl versus KO+AAV-Ctrl; ^#^*P*<0.05, ^##^*P*<0.01, *P*=0.0230 and 0.0091, KO+AAV-ASIC1a versus KO+AAV-Ctrl. (**k**) *n*=9–12. *P<0.05, *P*=0.0310, 0.0111 and 0.0320, NC-Ctrl versus ASIC1a-shRNA. (**p**) *n*=11–12. *P<0.05, **P<0.01, *P*=0.0328, 0.0418 and 0.0013, WT+AAV-NC-Ctrl versus WT+AAV-shRNA-DIO-ASIC1a*; ^#^*P*<0.05, ^##^*P*<0.01, *P*=0.0033, 0.0108 and 0.0016, WT versus CaMKII-Cre for AAV-shRNA-DIO-ASIC1a* injected, unpaired Student's *t*-test. (**b**) Group, F_(1,56)_=49.797, *P*<0.001; test day, F_(3,56)_=12.099, *P*<0.001; interaction, F_(3,56)_=5.884, *P*=0.002. (**e**) Group, F_(1,84)_=21.088, *P*<0.001; test day, F_(3,84)_=8.078, *P*<0.001; interaction, F_(1,84)_=6.222, *P*=0.001. (**g**) Group, F_(3,160)_=11.996, *P*<0.001; test day, F_(3,160)_=4.542, *P*=0.005; interaction, F_(9,160)_=1.842, *P*=0.066. (**k**) Group, F_(1,84)_=14.363, *P*<0.001; test day, F_(3,84)_=7.334, *P*<0.001; interaction, F_(3,84)_=3.187, *P*=0.028. (**p**) Group, F_(2,140)_=14.603, *P*<0.001; test day, F_(3,140)_=21.722, *P*<0.001; interaction, F_(6,140)_=1.796, *P*=0.105, two-way analysis of variance. (**d**) A diagram depicting drug diffusion. (**i**,**m**) Schematics of AAV vector constructs. (**j**,**o**) Representative images of western blots. (**n**) Example of insular mCherry expression in mice infected with AAV-shRNA-DIO-ASIC1a* (**m**). The slices were counterstained with DAPI. DIO, double-floxed inverted orientation.

To establish insular cortex as the critical site of ASIC1a action on CTA extinction, we again employed the conditional *ASIC1a*^*flox/flox*^ line as described above. Strikingly, AAV-Cre injection (Fig. 5a) resulted in greatly diminished CTA extinction in *ASIC1a*^*flox/flox*^ mice as compared with AAV-Ctrl injection (Fig. 5b). By contrast, the injection of AAV-Cre in hippocampal CA1 region did not alter CTA extinction (Supplementary Figs 10a–d and 13g). These results support a specific role for insula-expressed ASIC1a in CTA extinction.

We then tested whether acute inhibition of ASIC1a in the insular cortex could affect CTA extinction. PcTx1 (10 pmol) or vehicle was injected bilaterally into insular cortices immediately after the two-bottle choice test on each day for three consecutive days beginning 3 days after the conditioning (Fig. 5c). The administration of PcTX1 (Fig. 5d; Supplementary Fig. 5c) significantly attenuated CTA extinction as compared with the vehicle (Fig. 5e). In contrast, injecting PcTX1 (5 pmol) into the hippocampal CA1 region had no effect on CTA extinction (Supplementary Fig. 10e–g).

Next, we tested whether expression of exogenous ASIC1a in insular cortices could rescue the diminished CTA extinction in *ASIC1a* KO mice, using the same approach as shown in Fig. 1i–m, except that CTA was induced 1 month after the AAV-mediated expression and extinction was triggered by the choice test on days 3–6 after CTA conditioning (Fig. 5f). As expected, AAV-ASIC1a restored CTA extinction in the ASIC1a null mice to a similar level as the WT (AAV-Ctrl injected), while AAV-Ctrl and AAV-ASIC1a-HIF mutant were ineffective (Fig. 5g). Therefore, functional ASIC1a in insular cortex is the critical missing component in the *ASIC1a* KO mice that supports CTA extinction.

As an alternative approach to eliminate ASIC1a expression, we generated an AAV construct that expresses a short hairpin RNA (shRNA) targeting ASIC1a, driven by the U6 promoter (Fig. 5i). After verification of its effectiveness in Chinese hamster ovary (CHO) cells (Supplementary Figs 11 and 13h), AAV-ASIC1a-shRNA or a negative control virus (AAV-NC-Ctrl) was injected in the insular cortices of WT mice (Fig. 5h). AAV-ASIC1a-shRNA markedly decreased ASIC1a protein levels in the insular cortex (*P*=5.651E−05, *n*=4–6; Fig. 5j; Supplementary Fig. 13a) and led to attenuation of CTA extinction as compared with AAC-NC-Ctrl injected animals (Fig. 5k). We also performed a cell-type-specific rescue experiment by expressing exogenous ASIC1a in excitatory neurons together with the ASIC1a shRNA (Fig. 5l). The AAV vector with ASIC1a shRNA also contained a double-floxed inverted orientation coding sequence for shRNA-resistant mCherry-2A-ASIC1a (reading as ASIC1a*), which is only expressed in the presence of Cre recombinase (Fig. 5m). When injected into insular cortices of CaMKII-Cre mice, in which Cre recombinase is expressed in pyramidal neurons of forebrain^45^, robust expression of mCherry (Fig. 5n) and ASIC1a (Fig. 5o; Supplementary Fig. 13b) was observed in a subpopulation of insular neurons. No expression of mCherry (Fig. 5n) or ASIC1a (Fig. 5o) was detected in WT mice injected with the same virus. The shRNA contained in this AAV (Fig. 5m) was still effective at inhibiting CTA extinction in WT mice, as evidenced by the retention of high aversion index (Fig. 5p). By contrast, in CaMKII-Cre mice, the virus resulted in CTA memory extinction to a similar extent as in control animals (Fig. 5p). Collectively, the above results establish a pivotal role of insular ASIC1a in extinction of taste aversion.

### ASIC1a acts through GSK3β in insular cortex

**Figure 6:**
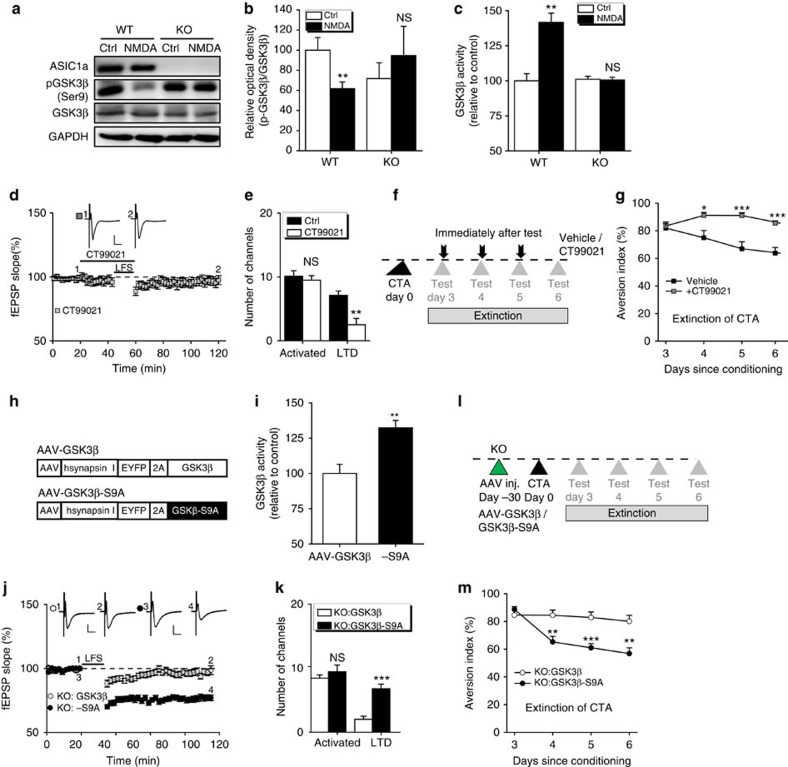
GSK3β signalling is involved in ASIC1a-mediated insular LTD induction and CTA memory extinction. (**a**,**b**) Representative images (**a**) and quantification (**b**) of western blots. *n*=6. **P<0.01, NS, not significant, *P*=0.0021 and 0.5035, Ctrl versus NMDA, unpaired Student's *t*-test. (**c**) GSK3β activities. *n*=5. NS, not significant, **P<0.01, *P*=0.0011 and 0.8486, Ctrl versus NMDA, unpaired Student's *t*-test. (**d**,**j**) Time courses of fEPSP slope changes in response to LFS for insular cortical slices prepared as indicated. Scale bar, 100 μV, 10 ms. (**e**,**k**) Summary of number of activated channels per slice and those that showed LTD. NS, not significant, **P<0.01, ***P<0.001, (**e**) *P*=0.6266 and 0.0014, *n*=6–9 slices per 6–8 mice; (**k**) *P*=0.4077 and 0.0001, *n*=7–8 slices per 4–5 mice, compared between two groups, unpaired Student's *t*-test. The Ctrl data in **e** were taken from Fig. 1d and regraphed for comparison. (**f**,**l**) The behavioural protocols used for **g**,**m**, respectively. (**g**,**m**) Time courses of aversion indices over a 4-day period for animals subjected to the CTA extinction protocol. *n*=6–10. *P<0.05, ***P<0.001, *P*=0.0101, 0.0003 and 0.0001 (**g**), 0.0055, 0.0009 and 0.0025 (**m**), compared between the two treatment groups, unpaired Student's *t*-test. (**g**) Group, F_(1,80)_=40.479, *P*<0.001; test day, F_(3,80)_=2.452, *P*=0.07; interaction, F_(3,80)_=4.109, *P*=0.009. (**m**) Group, F_(1,52)_=33.279, *P*<0.001; test day, F_(3,52)_=9.134, *P*<0.001; interaction, F_(3,52)_=6.084, *P*=0.001, two-way analysis of variance. (**h**) Schematics of AAV vector constructs. (**i**) Injection of AAV-GSK3β-S9A increased GSK3β enzymatic activity in lysates from insular cortices as compared with the injection of AAV-GSK3β. *n*=4–5. **P<0.01, *P*=0.0057, unpaired Student's *t*-test.

We further examined the key signalling pathway involved in insular LTD and CTA extinction. NMDAR-LTD in insular cortex requires protein phosphatase 1/2A (ref. 39), which was shown to regulate hippocampal NMDAR-LTD induction via glycogen synthase kinase-3β (GSK3β)^46^,^47^. Thus, we measured whether GSK3β was activated by stimulating NMDAR in insular cortex and whether such an effect required ASIC1a. Insular cortical slices were treated with NMDA (50 μM, 3 min). Western blotting revealed GSK3β dephosphorylation at Ser-9, that is, activation on NMDA stimulation of insular cortices from WT, but not *ASIC1a* KO mice (Fig. 6a,b; Supplementary Fig. 13c). In addition, an enzymatic assay showed that NMDA increased GSK3β activities only in samples from WT, but not those from *ASIC1a* KO mice (Fig. 6c). Therefore, NMDAR stimulation of insular cortex activates GSK3β in an ASIC1a-dependent manner.

We then applied a selective GSK3β inhibitor (CT99021, 1 μM)^48^,^49^ to insular cortical slices before and during LFS delivery. While CT99021 treatment for up to 20 min had no effect on basal excitatory synaptic transmission (Fig. 6d), it disrupted the development of LFS–LTD (Fig. 6d), with the induction ratio reduced to 26.6±9.2% from 70.5±4.9% for untreated control slices (*P*=0.005; Fig. 6e). Moreover, bilateral injection of CT99021 (200 pmol) into insular cortices of WT mice immediately after each two-bottle choice test on days 3–6 after conditioning (Fig. 6f) strongly attenuated CTA extinction (Fig. 6g). Thus, insular GSK3β signalling is also essential for CTA extinction, in addition to LTD induction.

Next, we examined whether ectopic expression of a constitutively active form of GSK3β (Ser-9 mutated to Ala, S9A) in the insular cortex could rescue the impaired LTD and CTA extinction in the *ASIC1a* KO mice. Injection of AAV-GSK3β-S9A (Fig. 6h) resulted in the increased GSK3β activity as compared with AAV-GSK3β (Fig. 6i), which also successfully rescued LFS–LTD (Fig. 6j), with the induction ratio reached 71.8±4.2%, significantly higher than that achieved by AAV-GSK3β (22.6±4.8%, *P*=3.165E–06; Fig. 6k). Furthermore, AAV-GSK3β-S9A but not AAV-GSK3β (Fig. 6l) restored CTA extinction in the ASIC1a null mice (Fig. 6m). Therefore, a failure to activate GSK3β in insular cortex represents the major deficit in the *ASIC1a* KO mice that led to the diminished LTD and CTA extinction.

### Insular LTD is required for CTA extinction

**Figure 7:**
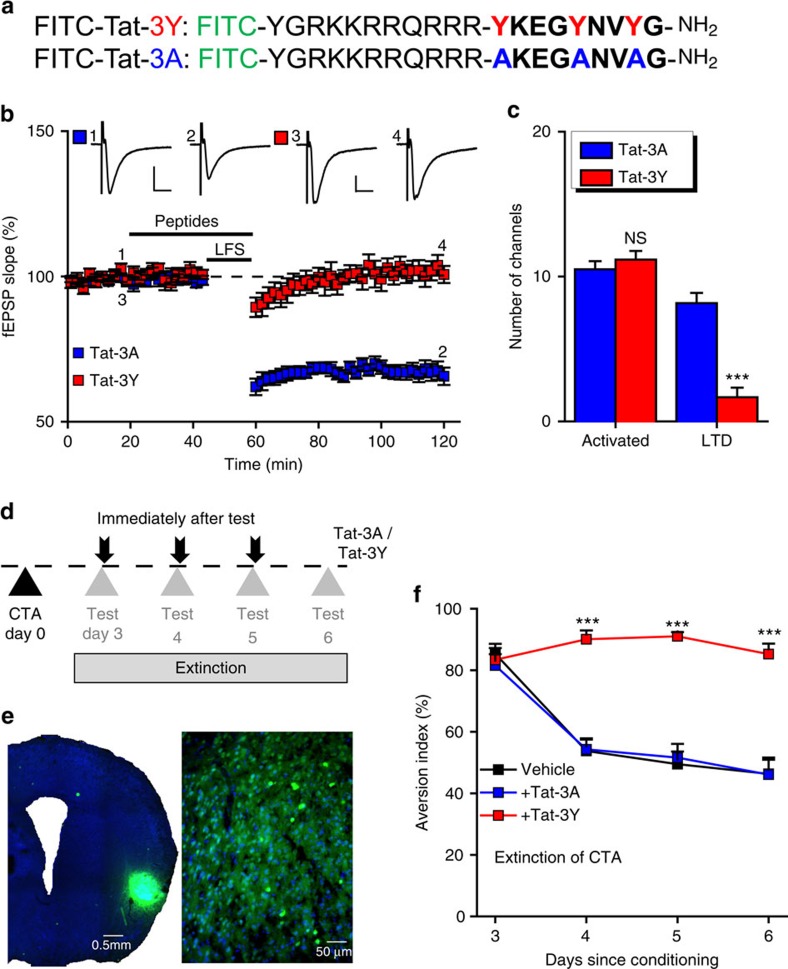
Insular LTD is necessary for CTA extinction. (**a**) Sequences of the interference peptide used for inhibiting AMPAR endocytosis and the corresponding nonfunctional control peptide, in which the critical tyrosine residues (Y, marked in red) were changed to alanines (A, printed in blue). (**b**) Acute application of Tat-3Y (1 μM, initiated at 20 min before the LFS protocol and sustained for a total duration of 35 min as indicated), but not Tat-3A (also 1 μM) blocked the LFS-evoked insular LTD. (**c**) Summary of number of activated channels per slice and those that showed LTD in insular synapses treated with Tat-3Y and Tat-3A. NS, not significant, ***P<0.001, *P*=0.4369 and 5.310E−05, *n*=6 slices per 5 mice each, Tat-3A versus Tat-3Y, unpaired Student's *t*-test. (**d**) Behavioural protocol used to test the effect of Tat-3Y and Tat-3A peptides on CTA memory extinction. The peptide (100 μM, 1 μl) or vehicle control (1 μl) was injected bilaterally into insular cortices of WT mice immediately after the two-bottle choice test on days indicated. (**e**) Images showing the presence of the fluorescent Tat-3Y peptide in the insular cortex of mouse brain (left) and in individual insular neurons at a high magnification (right). (**f**) Time courses of aversion indices over a 4-day period for animals subjected to the CTA extinction protocol. *n*=10–14 per group. ***P<0.001, *P*=3.063E−07, 3.312E−08 and 1.190E−05, vehicle versus Tat-3Y, unpaired Student's *t*-test. Group, F_(2,144)_=63.377, *P*<0.001; test day, F_(3,144)_=20.738, *P*<0.001; interaction, F_(6,144)_=7.315, *P*<0.001, two-way analysis of variance.

To establish that ASIC1a-dependent LTD in insular cortex is involved in CTA extinction, we investigated the behavioural consequence of blocking insular LTD *in vivo*. As a general mechanism of LTD^50^, AMPAR endocytosis can be blocked by either intracellular application of a GluA2 mimetic peptide, GluA2-3Y (refs 50, 51), or extracellular administration of a fluorescent infusion peptide FITC-Tat-GluA2-3Y (Tat-3Y; Fig. 7a), which contains a TAT peptide sequence derived from HIV to facilitate membrane penetration. Its negative control, FITC-Tat-GluA2-3A (Tat-3A; Fig. 7a) does not exert these effects. Application of Tat-3Y (1 μM), but not Tat-3A (1 μM), before and during LFS delivery, blocked LTD induction in insular cortical slices (Fig. 7b), with the induction ratios of 16.0±7.1% and 78.0±6.1% for Tat-3Y- and Tat-3A-treated slices (*P*=5.900E−05; Fig. 7c), respectively, indicating that GluA2-dependent AMPAR endocytosis represents a key step in insular LTD induction, similar to that in other brain regions^50^.

Finally, we assessed the effect of Tat-3Y (100 pmol) on CTA extinction (Fig. 7d). The successful delivery of the peptide was validated by the fluorescein isothiocyanate (FITC) fluorescence in insular cortex (Fig. 7e). Mice that received Tat-3Y displayed a striking deficiency in CTA extinction, as compared with those that received vehicle alone or Tat-3A (Fig. 7f). This result supports the notion that insular LTD is necessary for CTA extinction. Together, our data demonstrate that ASIC1a in the insular cortex confers a particular form of synaptic plasticity, which underlies its selective engagement in CTA extinction (Supplementary Fig. 12).

## Discussion

The foregoing data demonstrate that ASIC1a plays a critical role in insular LTD (Figs 1 and 2) and CTA extinction (Figs 4 and 5) through a mechanism that requires GSK3β signalling (Fig. 6). Multiple lines of evidence support a functional link between insular LTD and CTA extinction. First, in insular cortices of CTA-extinguished WT animals, AMPAR-mediated excitatory synaptic transmission was reduced and further LTD induction was severely hampered (Fig. 3). The reduced probability of LTD induction in insular cortical slices from CTA-extinguished mice could reflect an occlusion effect of already reduced synaptic strength or a decreased ASIC1a function due to channel desensitization, both of which might have occurred during CTA extinction learning. Second, inhibiting AMPAR endocytosis (Fig. 7), a general LTD mechanism^50^ or GSK3β signalling, critical for insular LTD (Fig. 6a–g) prevented CTA extinction. Third, disrupting ASIC1a not only inhibited CTA extinction, but also strongly decreased the probability of insular LTD induction, and both of these were rescued by expressing constitutively active GSK3β in insular neurons (Fig. 6h–m). Therefore, insular LTD, which is strongly dependent on ASIC1a and mediated via GSK3β, is involved in CTA memory extinction. The participation of a synaptic depression mechanism in insular cortex for CTA extinction is reminiscent of previous studies that implicate LTD to behavioural flexibility under other learning paradigms^52^,^54^.

The role of ASIC1a in insular LTD was unexpected because in other brain regions, ASIC1a has been shown to be important for LTP^6^,^17^,^20^,^21^. This disparity probably reflects the cognitive demand-dependent distinct eligibility traces^53^ for LTD or LTP in different synapses. It could also result from differential distribution and function of ASIC1a in excitatory and inhibitory neurons, as well as variations in ASIC subunit compositions and/or even binding partners in different brain regions. Therefore, ASIC1a has distinct roles in plasticity across brain regions, allowing it to exert complex behavioural effects.

Mechanistically, the following steps may underpin ASIC1a-mediated insular LTD. First, ASIC1a mediates Na^+^ and Ca^2+^ entry, which in turn increases cytosolic Ca^2+^ levels either directly through the ASIC or indirectly via voltage-gated Ca^2+^ channels. The latter are crucial players of both LFS- and DHPG-induced LTD in insular cortex^39^ and able to respond to the ASIC-mediated membrane depolarization. Since the diminished LTD in ASIC1a null mice was restored by re-introduction of WT, but not the pore-dead mutant of ASIC1a in insular neurons, the ionic conductance of ASIC1a is crucial for its role in insular synaptic depression. Second, as a necessary component of LFS-induced LTD in insular cortex^39^, the Ca^2+^-sensitive phosphatase 1/2A likely responds to the Ca^2+^ signal, resulting from ASIC1a activity and consequently activates GSK3β, a well-known key player of hippocampal LTD^47^. We showed that at least in NMDAR-LTD, ASIC1a was essential for the GSK3β activation and manipulating GSK3β functions altered insular LTD in the expected manner (Fig. 6). Third, through not fully characterized molecular events, GSK3β activation leads to AMPAR endocytosis^47^,^55^, a process that is dependent on the GluA2 subunit in multiple forms of LTD^50^,^51^. Indeed, a GluA2-derived interference peptide blocked LFS–LTD in insular cortex (Fig. 7a–c). Therefore, we propose a working model (Supplementary Fig. 12), in which postsynaptic ASIC1a regulates insular LTD induction through Ca^2+^-induced activation of phosphatase 1/2A and then GSK3β, either dependent or independent of NMDAR, which ultimately drives AMPAR internalization^56^ to cause synaptic depression.

A number of questions concerning this new form of synaptic plasticity warrant further investigation. First, how is ASIC1a involved in both LFS–LTD and DHPG-LTD in insular cortex? This may involve common mediators, such as protein interacting with C kinase 1 (PICK1), a well-characterized ASIC-binding partner^57^ that has been implicated in NMDAR-58 and metabotropic glutamate receptor-mediated LTD^59^ in other brain regions, and voltage-gated Ca^2+^ channels, previously shown to be required for both forms of LTD in insular cortex^39^. It remains to be determined if and how ASIC1a is functionally coupled to PICK1 or voltage-gated Ca^2+^ channels to confer the induction of both forms of LTD. Second, disrupting ASIC1a only strongly reduced the probability of, but did not completely abolish, LTD induction by LFS or DHPG, without affecting the success rate of LTP induction by TBS. This suggests a modulatory (albeit very strong), rather than essential, role of ASIC1a in synaptic plasticity of insular cortex. The strong modulatory effect mirrors its role in hippocampal LTP inducibility^20^, despite in the opposite direction, indicating flexibility of ASIC1a in tuning neural plasticity at different synapses. Third, disrupting ASIC1a reduced but did not abolish short-term depression, which is often believed to involve presynaptic mechanisms^60^. This contrasts the postsynaptic localization of ASIC1a in central neurons^4^,^5^,^6^,^16^,^17^,^18^,^22^. Thus, the new synaptic role of ASIC1a revealed here will inspire additional studies to further elucidate mechanisms of ASIC1a regulation on synaptic plasticity in various brain areas.

Behaviourally, the ASIC1a-dependent insular LTD is involved in extinction learning of aversive taste memory. This novel mechanism fills a major gap in our knowledge about taste learning. Previously, NMDAR-mediated, experience-dependent modifications of synapse strength^29^,^32^,^33^, as well as transcriptional^61^ and translational^62^ machineries, at insular cortex, have been shown to be indispensable underpinnings for taste memory formation. However, the mechanism for CTA extinction was poorly understood^36^. It was thought that insular cortex is important for subserving CTA extinction via a dissociable molecular machinery from CTA learning, as pharmacological inhibition of either protein synthesis or β-adrenergic receptors, but not NMDAR in insular cortex blocked CTA extinction^36^. Our finding that ASIC1a selectively contributes to CTA extinction, but not acquisition or retention (Fig. 4) supports this notion and adds important new knowledge to the understanding of taste learning and memory.

## Methods

### Animals

Animal care and the experimental protocols were approved by the Animal Ethics Committee of Shanghai Jiao Tong University School of Medicine, Shanghai, China (permit number: DLAS-MP-ANIM.01-05). To reduce the experimental variability, age-matched littermate pairs resulting from heterozygous crossings were used for all experiments. All behavioural measurements were performed in awake, unrestrained, mice (male, 2–3 months old, C57BL/6J background). The conventional global *ASIC1a* KO mice^6^ were the generous gifts of Professor Michael J. Welsh (Howard Hughes Medical Institute, University of Iowa, Iowa City, IA, USA). The conditional *ASIC1a*^*flox/flox*^ mice^19^ were graciously provided by Professor Cheng-Chang Lien (National Yang-Ming University, Taiwan). The *CaMKII-Cre* mice^45^ were kindly provided by Professor Joe Z. Tsien (Georgia Regents University, Augusta, GA, USA). All behavioural measurements were performed by raters blinded to the genotype and treatment. Three to four animals were housed per cage and maintained on a 12 h light/dark cycle with food and water *ad libitum* except where indicated otherwise. All experimental manipulations were performed during the light-on phase of the cycle in accordance with the institutional guidelines.

### Unconditioned taste preferences

The unconditioned taste preferences were tested according to a previous report^44^ with modifications. Full details of unconditioned taste preferences are in Supplementary Methods Online.

### CTA test

The CTA tests were performed as described previously^44^ with modifications, where the conditioned taste stimulus (sodium saccharin solution) was paired with an aversive US (LiCl injection; Supplementary Fig. 9a). Mice were adapted for ∼1 week in individual cages, in which they had *ad libitum* access to food, but restricted (from 9:00 to 9:30 a.m.) access to water presented in two bottles. Water intake during the 30 min drinking interval was recorded for each mouse. At the end of the adaptation phase, each mouse reliably consumed >1 g of water during the 30 min drinking period. On the day of conditioning, the mouse was allowed to drink only 0.5% sodium saccharin solution (0.5% w/v) (Sigma-Aldrich) provided in both bottles during the 30 min period. To induce malaise, each mouse in the conditioned group was given an intraperitoneal injection of LiCl (0.3 M, 2% of body weight, Sigma-Aldrich) 40 min after removal of the drinking bottles. After LiCl injection, the animal was visibly unwell and ceased all activity for several hours. On the test day, two bottles were inserted into each cage simultaneously, one containing water and the other containing the sodium saccharin solution. The placement of saccharin bottles with reference to water bottles was counterbalanced. Fluid consumption was determined by weighing both bottles before and after drinking. An aversion index for sodium saccharin was calculated as follows: aversion index (in %)=(water intake (in grams))/(sodium saccharin intake (in grams)+water intake (in grams)) × 100%. On days 1, 3, 7, 14 and 28 after conditioning, the two-bottle choice test was administered to determine the degree of CTA acquisition and retention (Fig. 4a,b). Animals were given water (but no sodium saccharin) during the 30-min drinking period before the test day. To determine the degree of recent CTA extinction, the two-bottle choice test was repeated for seven consecutive days beginning one day after conditioning (Fig. 4c). To evaluate the degree of remote CTA extinction, the two-bottle choice test was performed for seven consecutive days beginning on the 15th day after conditioning, before which the mice were given water only (Fig. 4d). For assessment of extinction following acute genetic or pharmacological manipulation (Figs 5, 6, 7; Supplementary Fig. 10), 3 days after CTA training, mice were given the two-bottle choice test, and the conditioned mice were tested once a day for four consecutive days. To fully extinguish CTA, the conditioned animals were allowed to drink only the saccharin solution (extinguished) during the 30-min drinking period for 7 days, whereas the control animals were given water only (CTA) to maintain the taste aversion (Fig. 3a). After that, the aversion index was obtained by subjecting the mice to the two-bottle choice test.

### Cell culture and electrophysiological recordings

The complementary DNA (cDNA) of mouse ASIC1a (GenBank accession: NM_009597.1) was expressed in CHO cells by transient transfection as reported previously^63^. Whole-cell patch-clamp electrophysiological recordings in cultured cells were conducted using the Molecular Devices (Foster City, CA, USA) system (Axoclamp 200B, Digidata 1440, pClamp 10). In most experiments, 70–90% of the series resistance was compensated. Voltage-clamp (with the holding potential of –60 mV) recordings were performed as described previously^63^. Full details are in Supplementary Methods Online.

### Brain slice preparation and patch-clamp recordings

Experiments were performed on insular slices from WT and mutant mice as described previously^25^,^39^,^52^ with minor modifications. Briefly, after decapitation, the mouse brain was quickly removed and placed in well-oxygenated (95% O_2_/5% CO_2_, v/v) ice-cold artificial cerebrospinal fluid (aCSF) containing (in mM): 125 NaCl, 2.5 KCl, 12.5 D-glucose, 1 MgCl_2_, 2 CaCl_2_, 1.25 NaH_2_PO_4_ and 25 NaHCO_3_ (pH 7.35–7.45). Three coronal insular slices (300 μm thick) were obtained at the level of the corpus callosum connection with a vibratome (Leica VT 1000S) and incubated at 30±1 °C in oxygenated aCSF for at least 1 h before being transferred to a recording chamber placed on the stage of an Olympus microscope (BX51WI). The placement of individual slice was observed using an infrared-differential interference contrast video monitor. The slices were continuously perfused with well oxygenated aCSF at room temperature during all electrophysiological studies. EPSCs were recorded from superficial layer (layer II and III) pyramidal neurons with an Axon 200B amplifier (Molecular Devices), and the stimulations were delivered with a bipolar tungsten stimulating electrode placed in the deep layer (layer V and VI). AMPAR-mediated EPSCs were induced by repetitive stimulations at 0.03 Hz, with the neuron voltage-clamped at –70 mV except where indicated otherwise. The recording pipettes (3–5 MΩ) were filled with a solution containing (in mM): 132.5 caesium gluconate, 17.5 CsCl, 2 MgCl_2_, 0.5 EGTA, 10 HEPES, 4 Mg-ATP and 5 QX-314 chloride (280–300 mOsm, pH 7.2 with CsOH). Spiking activity was measured with an internal solution containing (in mM): 145 potassium gluconate, 5 NaCl, 10 HEPES, 2 MgATP, 0.1 Na_3_GTP, 0.2 EGTA and 1 MgCl_2_ (280–300 mOsm, pH 7.2 with KOH). Pyramidal neurons were identified based on their ability to exhibit spike frequency adaptation in response to prolonged depolarizing current injection^25^,^26^ (Supplementary Fig. 2a). Access resistance was 15–30 MΩ and only cells with a change in access resistance <20% were included in the analysis. The input–output relation of EPSCs was obtained at –70 mV (Supplementary Fig. 2e). The NMDAR-mediated EPSCs were recorded in the presence of 6-cyano-7-nitroquinoxaline-2,3-dione (CNQX 20 μM) and picrotoxin (100 μM) with the neuron voltage clamp at +40 mV (Supplementary Fig. 2j,k). The miniature EPSCs of superficial layer (layer II and III) pyramidal neurons in insular cortex were obtained at –70 mV in the presence of tetrodotoxin (0.3 μM) and picrotoxin (100 μM) without stimulation (Supplementary Fig. 2g–i) and analysed using MiniAnalysis (Synaptosoft).

### Preparation of multi-electrode probe

A commercial 64-channel multisite recording system (MED64; Alpha-Med Sciences) was used for extracellular field potential recordings^38^,^39^. Full details are in Supplementary Methods Online.

### Multi-channel field potential recordings

Experimental procedures for multi-channel field potential recordings on insular cortical slices were similar to those described previously^38^,^39^. After incubation, one slice was positioned on the MED64 probe in such a way that the area of insular cortex was entirely covered by the recording dish mounted on the stage of an inverted microscope (CKX41; Olympus). The location of the insular slice relative to the probe (Fig. 1a,b) followed the anatomical atlas^64^. Once the slice was settled, a fine mesh anchor (Warner Instruments, Harvard Apparatus) was carefully positioned to ensure slice stabilization during recording. The slice was continuously perfused with oxygenated (95% O_2_/5% CO_2_, v/v), fresh standard extracellular solution containing (in mM): 124 NaCl, 2.5 KCl, 10 D-glucose, 1 MgSO_4_, 2 CaCl_2_, 1 NaH_2_PO_4_ and 25 NaHCO_3_, at a rate of 2 ml min^−1^ with the aid of a peristaltic pump (Minipuls 3; Gilson) throughout the experimental period. The perfusion temperature was maintained at 30±1 °C.

After the slice had been allowed to recover for 20 min, one of the 64 available planar microelectrodes was selected for stimulation by visual observation through a charge-coupled device camera (DP70; Olympus) connected to the inverted microscope (Fig. 1b). For test stimulation, monopolar, biphasic constant-current pulses (0.2 ms in duration) generated by the data acquisition software (MOBIUS; Panasonic Alpha-Med Sciences) were applied to the deep layer of the insular slice at 0.008 Hz. The fEPSPs evoked at the remaining sites were amplified by a 64-channel amplifier, displayed on the monitor screen and stored on the hard disk of a microcomputer for offline analysis. After selection of the best stimulation site and stabilization of the baseline synaptic responses, an input–output curve was first determined by using the measurements of the fEPSP slope or the number of activated channels (output) in response to a series of ascending stimulation intensities from 8 to 24 μA in 2 μA steps (input).

For LTP induction, after the baseline synaptic responses were stabilized for at least 1 h, a TBS protocol (10 bursts at 5 Hz, 4 pulses at 100 Hz for each burst) was given at a stimulation intensity that was adjusted to elicit 40–60% of the maximal response. After TBS, the test stimulus was repeatedly delivered once every min for at least 3 h to allow long-term monitoring of insular LTP induction and maintenance. For LTD induction, the intensity of the test stimulus was adjusted to elicit 40–60% of the maximum response. Stable baseline responses were then recorded for at least 20 min before delivery of the LFS protocol (1 Hz, 900 pulses, with the same intensity) to the deep layer of the insular slice. After LFS, the test stimulus was repeatedly delivered once every min for at least 1 h to monitor any change in LTD expression. For chemically evoked LTD, after stabilization of the baseline responses, DHPG (100 μM, Tocris Bioscience) was applied for 20 min through perfusion, which was followed by washout for at least 50 min. To test the effects of channel inhibitors on LTP induction (Fig. 2g), the ASIC1a inhibitor psalmotoxin 1 (PcTX1, 100 nM, Peptide Institute) was applied from 20 min before till 20 min after the TBS delivery. For LFS–LTD, PcTX1 was applied from 15 min before LFS and maintained continuously during the LFS (Fig. 1g). To test the effects of GSK3β inhibition on LTD induction, a specific inhibitor CT99021 (1 μM, Sigma-Aldrich) was applied from 20 min before the LFS protocol and sustained for a total duration of 35 min as indicated (Fig. 6d). To test the effects of blockade of GluA2 endocytosis on LTD induction, Tat-3Y or Tat-3A (both 1 μM, GL Biochem Ltd) was applied from 20 min before the LFS protocol and sustained for a total duration of 35 min as indicated (Fig. 7b). To test the effects of increasing pH buffering capacity on LTD induction, a well oxygenated (85% O_2_/15% CO_2_, v/v) and highly-pH-buffering extracellular solution containing (in mM): 79 NaCl, 2.5 KCl, 10 D-glucose, 1 MgSO_4_, 2 CaCl_2_, 1 NaH_2_PO_4_ and 70 NaHCO_3_, pH 7.35–7.45, was applied from 20 min before the LFS protocol and sustained for a total duration of 35 min as indicated (Supplementary Fig. 6).

All multi-channel electrophysiological data were analysed off-line by the MED64 Mobius software. For quantification of LTP and LTD data, the slope of fEPSPs was measured and normalized to that of the initial one and expressed, as percentage change from the baseline level. The numbers of activated channels (that is, with the fEPSP amplitude going over −20 μV) versus LTP-showing (fEPSP slope increased by at least 20% of baseline) or LTD-showing (fEPSP slope depressed by at least 15% of baseline) channels were counted and the induction ratios of LTP/LTD calculated as follows^38^,^39^: (number of LTP- or LTD-showing channels/number of all activated channels) × 100%.

### Generation of AAV vectors

AAV vectors (pAAV-MCS) carrying the full length of WT or mutant (^32^HIF^34^AAA) mouse *ASIC1a* cDNA or the full length of WT or mutant (S9A) human *GSK3β* were constructed with the coding sequence of EYFP fused to the N terminus of *ASIC1a* or *GSK3β* using the ‘self-cleaving' 2A-peptide, which can separate two proteins apart during the protein translation^42^. The transcription of the fusion protein EYFP-2A-ASIC1a, EYFP-2A-ASIC1a-^32^HIF^34^AAA, EYFP-2A-GSK3β or EYFP-2A-GSK3β-S9A was driven by human synapsin I (hsynapsin I) promoter to specifically limit the expression to neurons. The construct was then packaged into AAV2/8 chimeric virus with AAV8 capsids and AAV2 ITR (inverted terminal repeat) element. Once expressed *in vivo*, *EYFP-2A* and *ASIC1a* (or *GSK3β*) should be expressed separately and leave only a single proline residue at the N terminus of *ASIC1a* (or *GSK3β*). The control AAV vector (AAV-Ctrl) was constructed by sub-cloning the EGFP-coding sequence to the pAAV-MCS vector following the hsynapsin I promoter. The similar strategy was used to construct the AAV vector for expression of Cre recombinase driven by the hsynapsin I promoter.

To make the shRNA constructs, oligonucleotides that contained 21-base sense and antisense sequences targeting mouse *ASIC1a* (GenBank accession: NM_009597.1, sense sequence, 5′-GGACATTCAGCAAGATGAATA-3′) were connected with a hairpin loop followed by a poly(T) termination signal. For initial testing of the efficacy of the shRNA, the full-length *ASIC1a* cDNA was transfected into CHO cells together with the negative control (NC Ctrl, with sense sequence: 5′-GTTCTCCGAACGTGTCACGT-3′) or shRNA plasmid using the vector pGLV1-U6-GFP (Genepharm), from which the levels of ASIC1a protein expression and channel function were then assessed by western blotting and electrophysiological recordings, respectively. To prepare protein samples from CHO cells, the cells were washed with phosphate-buffered saline at 48 h after co-transfection and lysed in the lysis buffer. The re-suspended lysates were incubated on ice for 30 min and centrifuged at 13,000*g* at 4 °C for 15 min. Then the supernatants were collected for western blotting. Finally, the AAV vectors engineered to express the validated NC-Ctrl and ASIC1a shRNA were constructed under the promoter control of U6, a Pol III promoter that selectively drives the expression of small RNAs (Fig. 5i).

To achieve a specific expression of *ASIC1a* in pyramidal neurons in *CaMKII-Cre* mice, AAV vectors with a double-floxed inverted open reading frame^65^ under the control of a non-specific promoter, EF1α (elongation factor 1α), were used. In brief, the FLAG-tagged shRNA-resistant *ASIC1a* was cloned and inserted between loxP (open triangles in Fig. 5m) and lox2722 (shaded triangles in Fig. 5m) sites in the reverse orientation. The resulting double-floxed reverse FLAG-ASIC1a-2A-mCherry was cloned into the AAV vector-expressing ASIC1a shRNA as illustrated in Fig. 5m.

### Delivery of AAV

For virus injection, mice at the age of 4–8 weeks were anaesthetized and placed in a stereotaxic frame (RWD Life Science). Viruses (the titres of all vectors>1.0 × 10^12^ viral genome-containing particles per ml) were injected bilaterally into the insular cortex. The stereotaxic coordinates according to the mouse brain atlas^64^ were: anteroposterior, +0.98 mm; lateral, ±3.65 mm; dorsoventral, and –3.60 mm. One injection (1 μl) was made on each side of the insular cortex using microelectrodes connected with a microinjector pump (KDS 310, KD Scientific) at a rate of 0.1 μl min^−1^. Microelectrodes were left *in situ* for an additional 10 min to allow the injectant to diffuse. Mice were allowed to recover for 4 weeks before behavioural analysis and the injection sites were examined at the end of the experiments. Brain slices from animals injected with the viruses were examined directly using fluorescent microscopy.

### Surgical procedures and drug microinjection

Animals anaesthetized with 1% pentobarbital sodium and placed in a stereotaxic apparatus (RWD Life Science) were implanted bilaterally with a 26-Gauge guide cannula aimed at 0.50 mm above the targeted region on each side following the coordinates^64^ below: insular cortex: anteroposterior, +0.98 mm; lateral, ±3.55 mm; dorsoventral, –3.20 mm; hippocampal CA1: anteroposterior, –2.06 mm; lateral, ±1.65 mm; dorsoventral, –0.30 mm. The cannulas were positioned in place with acrylic dental cement and secured with skull screws. A stylus was placed in the guide cannula to prevent clogging. Animals were allowed to recover from surgery for a week before experimental manipulations. For microinfusion, the stylus was removed from the guide cannula, and a 30-Gauge infusion cannula (extending 0.50 and 1.00 mm from the tip of the guide cannula for targeting insular cortex and hippocampal CA1, respectively) was inserted. The infusion cannula was connected via PE20 tubing to a microsyringe driven by a microinfusion pump (KDS 310, KD Scientific). PcTX1 (10 μM in aCSF, 1 and 0.5 μl per side for insular cortex and CA1, respectively, Peptide Institute), CT99021 (200 μM in aCSF, 1 μl per side for insular cortex, Sigma-Aldrich), Tat-3Y (100 μM in aCSF, 1 μl per side for insular cortex, GL Biochem Ltd), Tat-3A (100 μM in aCSF, 1 μl per side for insular cortex, GL Biochem Ltd) or their respective vehicles were microinfused into these regions. The injection sites were examined at the end of the experiments, and animals with incorrect diffusion scope were excluded from the data analysis.

### Real-time reverse transcription PCR

Insular cortices of WT and ASIC1a KO mice were dissected and total RNA was extracted using TRIzol reagent (Thermo Fisher Scientific). Four micrograms of total RNA were used as a template for cDNA synthesis and amplification with the SuperScript III First-Stand Synthesis System (Thermo Fisher Scientific) according to the manufacturer's instructions. Full details are in Supplementary Methods Online.

### Preparation of PSD fractions

The purification of the PSD fraction was performed based on the previous studies^66^,^67^ with some modifications. Full details are in Supplementary Methods Online.

### NMDA stimulation of insular cortical slices

NMDA stimulation of brain slices was performed as previously described^52^ with minor modifications. Insular cortical slices (300 μm) were incubated in aCSF for 1 h at 31 °C before NMDA application. One half of each slice was incubated with NMDA (50 μM) for 3 min at 31 °C, while the other half was maintained in aCSF without NMDA as the control. After incubation, the insular region from each slice was dissected and then homogenized in the lysis buffer on ice. The lysates were used for western blotting with a phosphor-GSK3β antibody that recognizes phosphorylation at Ser-9 or the GSK3β activity assay (see below).

### Western blotting

Protein samples from different regions of mouse brain, including insular cortex and other areas, were separated by SDS–polyacrylamide gel electrophoresis and transferred to polyvinylidene difluoride filters. The filters were incubated overnight at 4 °C with appropriate antibodies. Secondary antibodies conjugated to horseradish peroxidase were added to the filters and then visualized in ECL solution. The visualization was performed via the ImageQuant LAS 4000 mini Molecular Imaging System (GE Healthcare Life Sciences), and the Image J software (NIH) was used for the analysis of band intensity. Antibodies used were as follows: ASIC1a (1:500; Santa Cruz, sc-13905), β-actin (1:1,000; Chemicon, Cat # MAB1501), GAPDH (1:1,000; KangChen, Cat # KC-5G4), GluA1 (1:1,000; Epitomics, Cat # 3861-1), GluA2 (1:1,000; Epitomics, Cat # 3520-1), GluN1 (1:1,000; R&D Systems, Cat # PPS011B), GluN2A (1:1,000; Millipore, Cat # 07-632), GluN2B (1:1,000; Millipore, Cat # MAB5220), PSD-95 (1:1,000; Epitomics, Cat # 2366-1), pGSK3β-Ser9/pGSK3α-Ser21 (1:1,000; Cell Signaling Technology, Cat # 8566) and GSK3β (1:1,000; Cell Signaling Technology, Cat # 12456).

### GSK3β activity assay

The GSK3β Activity Assay Kit (GENMED) was used according to the manufacturer's instructions. The principle of the colorimetric method is based on the activity of GSK3β to phosphorylate the target sequence GPHRSTPESRAAV in the presence of ATP and then through reactions that involve pyruvate kinase and lactate dehydrogenase to oxidize NADH (reduced nicotinamide adenine dinucleotide) into NAD (nicotinamide adenine dinucleotide). The absorbance was read at 340 nm.

### Reagents

Most of the drugs and chemicals used in these experiments were purchased from Sigma-Aldrich unless where indicated otherwise. The lysis buffer used for protein extraction contained 20 mM Tris-Cl, pH 7.4, 150 mM NaCl, 1% Triton X-100, 1 mM EDTA, 3 mM NaF, 1 mM β-glycerophosphate, 1 mM Na_3_VO_4_ and 10% glycerol, with protease inhibitors and phosphatase inhibitors.

### Statistical analyses

No statistical methods were used to predetermine sample sizes, but the sample sizes used are similar to those generally employed in the field. The data were collected and processed randomly. All behavioural tests and analysis were blindly conducted. Variance was similar between the groups being statistically compared and data were normally distributed supporting the use of parametric statistics. Data were analysed with Student's *t*-test or two-way repeated-measures analysis of variance, followed by Fisher's least significant difference *post hoc* comparisons, where appropriate. *P*<0.05, represent significant differences. All summary values in the text and figures represent means±s.e.m. Data analyses were performed using SPSS statistical program version 10.0.

### Data availability

The authors declare that the data supporting the findings of this study are available within the article and its Supplementary Information files or from the authors on request.

## Additional information

**How to cite this article:** Li, W. G. *et al*. ASIC1a regulates insular long-term depression and is required for the extinction of conditioned taste aversion. *Nat. Commun.* **7,** 13770 doi: 10.1038/ncomms13770 (2016).

**Publisher's note**: Springer Nature remains neutral with regard to jurisdictional claims in published maps and institutional affiliations.

## Supplementary Material

Supplementary Information Supplementary Figures, Supplementary Methods and Supplementary References
