# Supplementary material for: ASIC1a regulates insular long-term depression and is required for the extinction of conditioned taste aversion
Source: Nat Commun. 2016 Dec 7;7:13770. doi: 10.1038/ncomms13770 (PMC5150990; doi:10.1038/ncomms13770)
Supplement: Supplementary Information — Supplementary Figures, Supplementary Methods and Supplementary References [file ncomms13770-s1.pdf]

Supplementary Figure 1 by Li *et al.*

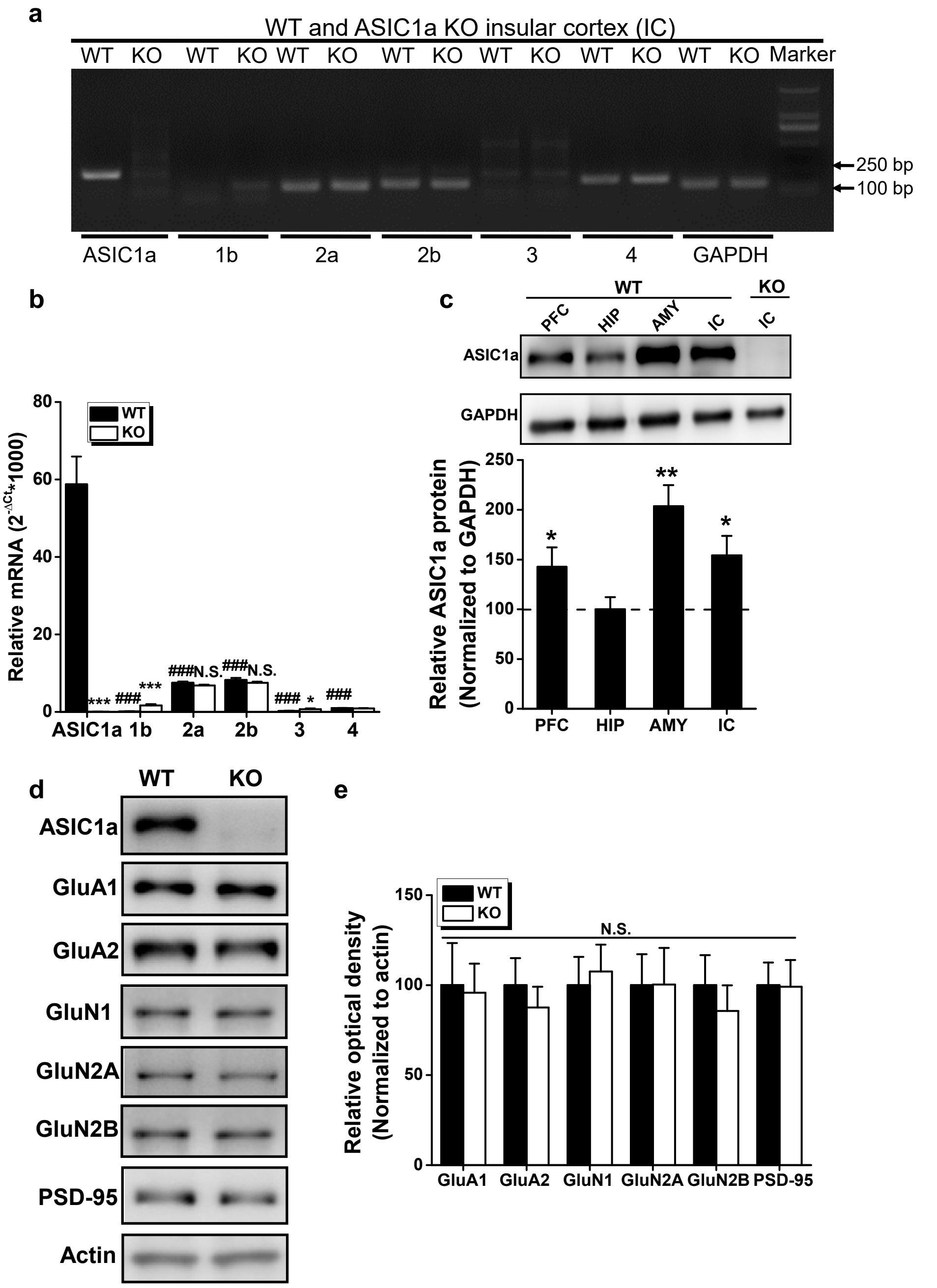

Supplementary Figure 1. ASIC1a is highly expressed in insular cortex but its ablation exerted a negligible effect on insular cortical synaptic protein compositions. (**a-c**) ASIC1a is highly expressed in insular cortex. (**a**) The mRNA levels of known ASIC isoforms in insular cortical tissues from WT and *ASIC1a* KO mice, as examined by RT-PCR. (**b**) Quantification of the mRNA levels using real time RT-PCR. n = 3 mice for each group. N.S., not significant, $^{***}P < 0.001$, P = 1.563E-09, 1.798E-05, 0.1020, 0.2366, 0.0255, and 0.4459 for comparison of the expression of ASIC1a, 1b, 2a, 2b, 3, and 4, receptively, in insular cortical tissues from WT *vs.* *ASIC1a* KO mice; $^{\#\#\#}P < 0.001$, P = 1.608E-09, 3.054E-08, 4.253E-08,

1.671E-09, and 2.214E-09 for comparison of the expression of *ASIC1b*, *2a*, *2b*, *3*, and *4*, respectively, with *ASIC1a* subtype in WT insular cortical tissues, unpaired Student's *t*-test. (**c**) ASIC1a protein levels in insular cortex (IC) and other brain regions. *Upper*, representative immunoblots showing ASIC1a protein expression in prefrontal cortex (PFC), hippocampus (HIP), amygdala (AMY), and IC. *Lower*, quantification of immunoblots as shown in the upper panel. Data represent optical density values normalized to that of hippocampus. n = 4 mice for each group. $^{*}P < 0.05$, $^{**}P < 0.01$, $P = 0.0363$, 0.0026, and 0.0326 for comparison of the expression of ASIC1a in prefrontal cortex, amygdala, and insular cortex, respectively, with that in the hippocampus (dashed line), paired Student's *t*-test. (**d, e**) Synaptic protein compositions in insular cortices of WT and ASIC1a null mice. (**d**) Representative immunoblots. (**e**) Pooled data from experiments as shown in (**d**). n = 5 each group. N.S., not significant, $P = 0.7999$, 0.3112, 0.6279, 0.9888, 0.3420, and 0.9532 for comparison of the expression of GluA1, GluA2, GluN1, GluN2A, GluN2B, and PSD-95, receptively, in insular cortices of WT *vs.* *ASIC1a* KO, unpaired Student's *t* test.

Supplementary Figure 2 by Li *et al.*

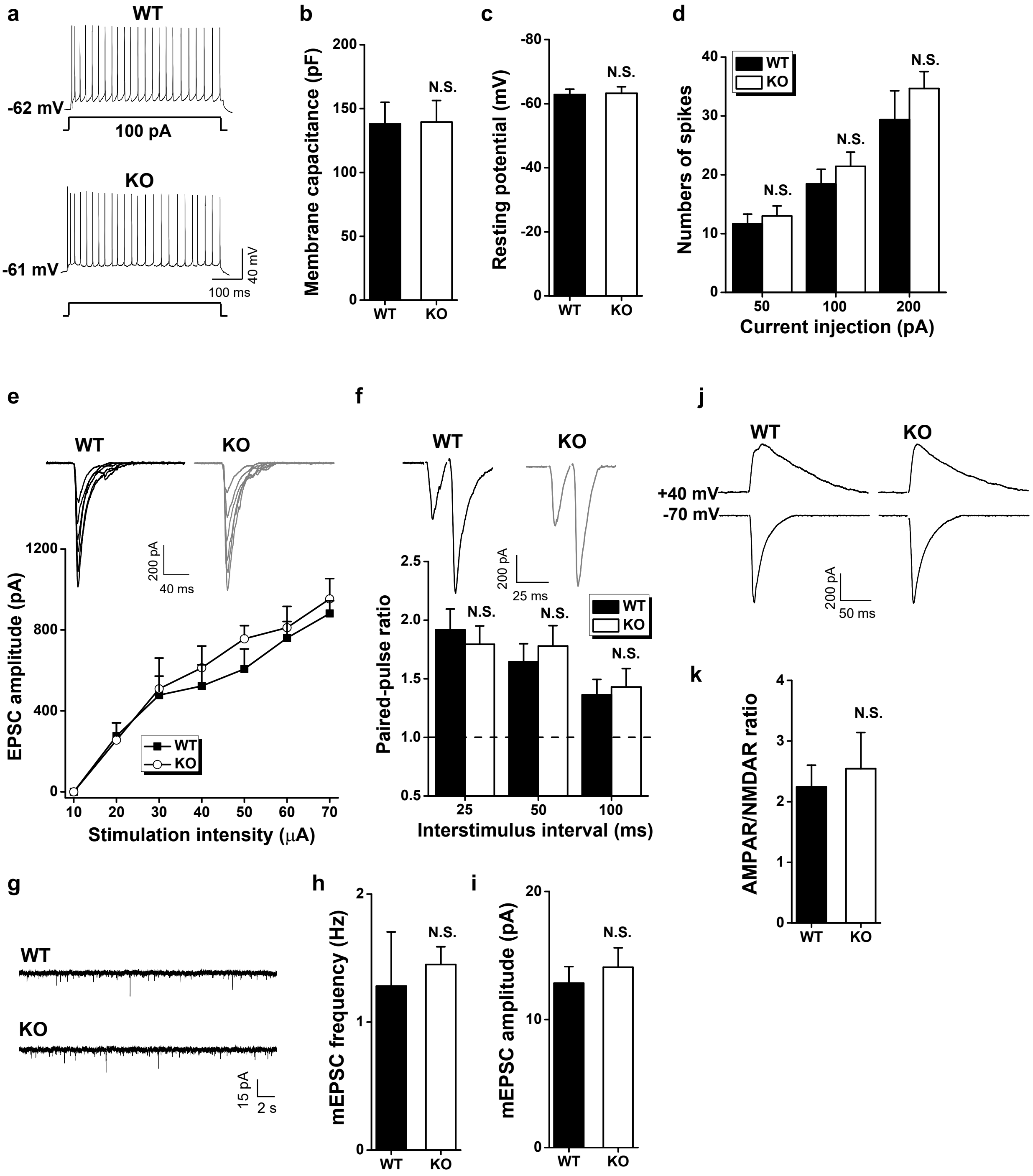

Supplementary Figure 2. Intrinsic electrophysiological and synaptic properties of insular cortices in *ASIC1a* KO mice are comparable to those of WT littermates. (**a-d**) Intrinsic membrane properties. (**a**) Representative traces showing voltage responses of pyramidal neurons in layer II-III of insular cortex to 500 ms injection of current of +100 pA. (**b-d**) Summary data (15 cells from 4 WT mice and 17 cells from 5 *ASIC1a* KO mice) for membrane capacitance (**b**), resting potential (**c**), and spike

frequency (**d**) of pyramidal neurons based on measurements exemplified in (**a**). N.S., not significant, *P* = 0.9144, 0.6510, 0.5700, 0.4860, and 0.4082 for comparison of membrane capacitance (**b**), resting potential (**c**), numbers of spikes (**d**) in response to injection of current of +50, +100, or +200 pA, respectively, in insular cortical slices from WT *vs. ASIC1a* KO mice, unpaired Student's *t* test. (**e-k**) Synaptic properties. (**e**) Input-output relationships of insular cortical neurons from WT and *ASIC1a* KO mice. Shown are representative traces (*upper*) and summary data (9 cells from 4 WT mice and 8 cells from 5 *ASIC1a* KO mice, *lower*). Stimulation artefacts were removed for clarity. *P* = 0.8594, 0.8639, 0.5616, 0.2963, 0.7050, and 0.5352 for comparison of EPSC amplitude in response to the stimulation by 20–60 μA (10 μA per step), respectively, in insular cortical slices from WT *vs. ASIC1a* KO mice, unpaired Student's *t* test. (**f**) Paired-pulse ratios of insular cortical neurons from WT and *ASIC1a* KO mice. Shown are representative traces (interstimulus interval = 25 ms, *upper*) and summary data (8 cells from 5 WT mice and 10 cells from 8 *ASIC1a* KO mice, *lower*). N.S., not significant, *P* = 0.6520, 0.6270, and 0.7520 for comparison of paired pulse ratios in response to 25, 50, 100 ms interstimulus intervals, respectively, in insular cortical slices from WT *vs. ASIC1a* KO mice, unpaired Student's *t* test. (**g-i**) Representative traces of mEPSCs recorded from insular cortical neurons of WT and *ASIC1a* KO mice (**g**) and summary (14 cells from 5 WT mice and 12 cells from 9 *ASIC1a* KO mice) of frequency (**h**) and amplitude (**i**). N.S., not significant, *P* = 0.7268 and 0.5353 for comparison of frequency (**h**) and amplitude (**i**) of mEPSC, respectively, in insular cortical slices from WT *vs. ASIC1a* KO mice, unpaired Student's *t* test. (**j, k**) Representative traces of AMPAR (*lower*) and NMDAR (*upper*) currents recorded from insular cortical neurons of WT (*left*) and *ASIC1a* KO (*right*) mice (**j**) and summary (**k**) of AMPAR/NMDAR ratios for 8 cells from 6 WT mice and 4 cells from 4 *ASIC1a* KO mice. Stimulation artefacts were removed for clarity. N.S., not significant, *P* = 0.6557 for comparison of AMPAR/NMDAR ratios in insular cortical slices from WT *vs. ASIC1a* KO mice, unpaired Student's *t* test.

Supplementary Figure 3 by Li *et al.*

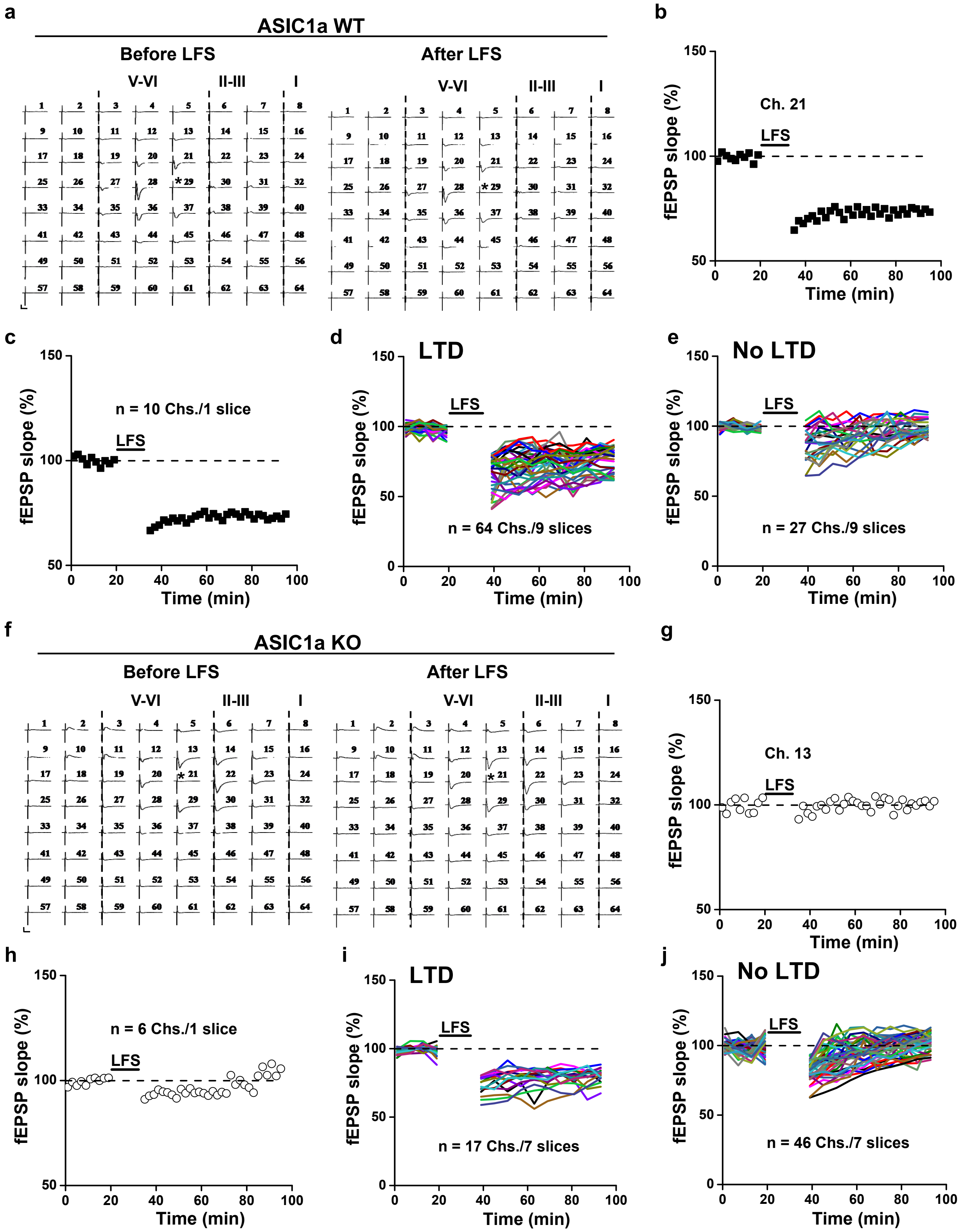

Supplementary Figure 3. Multi-channel recordings of LFS-evoked LTD in insular cortical slices from WT and *ASIC1a* KO mice. (a-e) WT mice. (**a**) The

64-channel overview of multisite synaptic responses recorded at baseline *(left*) and 60 min after LFS (1 Hz, 900 pulses, *right*) in an insular cortical slice prepared from a WT mouse. Asterisks indicate the stimulated channel (Ch. 29). Scale bar: 100 μV, 10 ms. (**b**) Time course of fEPSP slope changes of a representative channel that exhibited LTD (Ch. 21). (**c**) Time course of mean fEPSP slopes of all 10 activated channels in the slice shown in (**a**). (**d, e**) Pooled data of 64 channels that exhibited LTD (**d**) and 27 channels that failed to show LTD (**e**) from a total of 9 slices analyzed for 8 WT mice. (**f-j**) *ASIC1a* KO mice. (**f**) The 64-channel overview of multisite synaptic responses recorded at baseline (*left*) and 60 min after LFS (*right*) in an insular cortical slice prepared from an *ASIC1a* KO mouse. Asterisks indicate the stimulated channel (Ch. 21). Scale bar: 100 μV, 10 ms. (**g**) Time course of fEPSP slope changes of a representative channel that showed no LTD (Ch. 13). (**h**) Time course of mean fEPSP slopes of all 6 activated channels in the slice shown in (**f**). (**i, j**) Pooled data of 17 channels that exhibited LTD (**d**) and 46 channels that showed no LTD (**e**) from a total of 7 slices analyzed for 7 *ASIC1a* KO mice. Note, for display purposes, some channels are not shown due to their extraordinarily high magnitude of acute depression or large fluctuation.

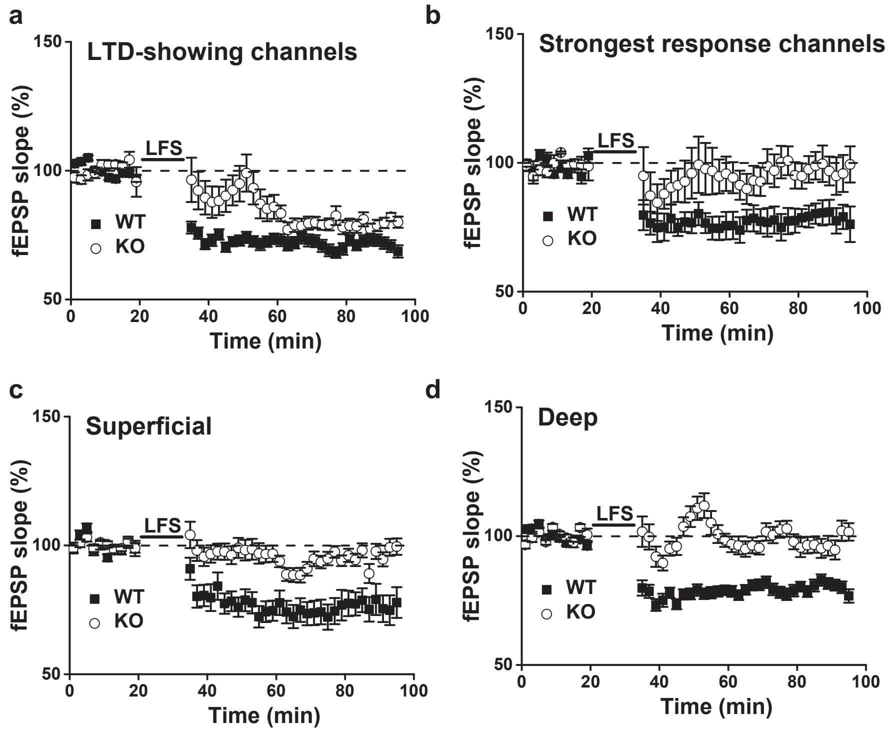

Supplementary Figure 4. Time courses of fEPSP slope changes in insular cortical slices from WT and *ASIC1a* KO mice, analyzed based on different criteria of data selection. (**a**) Only channels that developed LTD are included. WT: n = 64; *ASIC1a* KO: n = 17. *P* = 0.0677. (**b**) Only channels with the strongest fEPSP response in individual slices are included. WT: n = 9; *ASIC1a* KO: n = 7. *P* = 0.0166. (**c**) All channels that measured from the superficial layer are counted. WT: n = 28; *ASIC1a* KO: n = 37. *P* = 0.0003. (**d**) All channels that measured from the deep layers are counted. WT: n = 63; *ASIC1a* KO: n = 48. *P* = 3.314E-07. LFS was 1 Hz, 900 pulses. Statistical analysis between WT *vs.* *ASIC1a* KO was performed by comparing the average slope of fEPSP responses over a 10-min period (85–95 min). Statistical significance was determined using unpaired Student's *t* test.

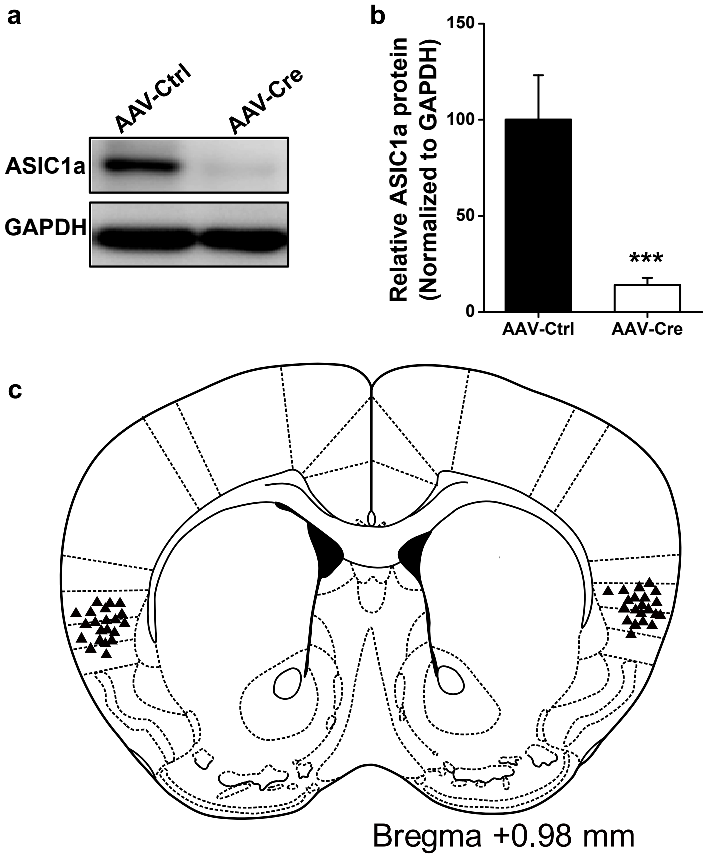

Supplementary Figure 5 Characterization of the effectiveness in insular cortex by genetic or pharmacological approaches. (**a, b**) Analysis of genetic manipulation of ASIC1a in insular cortex. Injection of AAV-Cre, but not AAV-Ctrl, into the insular cortex of floxed ASIC1a (*ASIC1a*$^{flox/flox}$) mice reduced ASIC1a protein expression. (**a**) Representative immunoblots. (**b**) Pooled data from experiment exemplified in (**a**). n = 3 mice for each group. $^{***}P < 0.001$, $P = 2.226E\text{-}05$, AAV-Ctrl v*s*. AAV-Cre, unpaired Student's *t*-test. (**c**) Infusion sites of insular cortex at the section of Bregma +0.98 mm from the experiment shown in **Fig. 5e**.

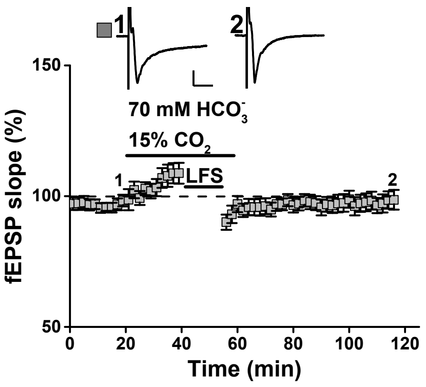

Supplementary Figure 6. Increasing pH buffering capacity of extracellular solution by increasing HCO_3^- and CO_2 prevented LTD induction in insular cortex. Time course of fEPSP slope changes showing that increasing the buffering capacity abolished LTD induction in insular synapses (n = 56 activated channels from 7 slices / 6 WT mice). Inset: representative fEPSP traces at the time points indicated by numbers in the graph. Scale bar: 100 μV, 10 ms.

Supplementary Figure 7 by Li *et al.*

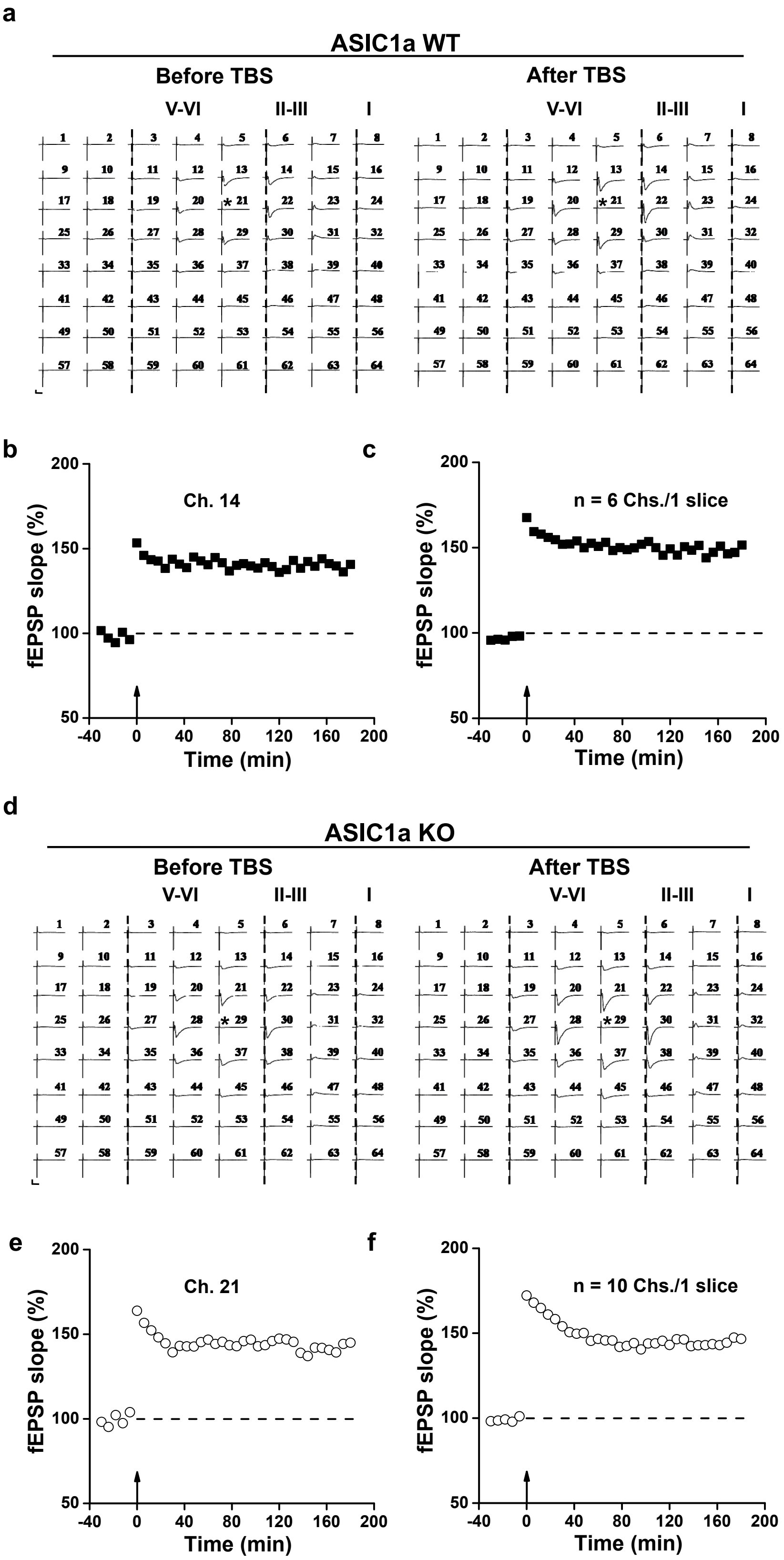

Supplementary Figure 7. Multi-channel recordings of TBS-evoked LTP in

insular cortical slices from WT and *ASIC1a* KO mice. (**a-c**) WT mice. (**a**) The 64-channel overview of multisite synaptic responses recorded at baseline (*left*) and 3 hr after TBS (10 bursts at 5 Hz, 4 pulses at 100 Hz for each burst, *right*) in an insular cortical slice prepared from a WT mouse. Asterisks indicate the stimulated channel (Ch. 21). Scale bar: 100 μV, 10 ms. (**b**) Time course of fEPSP slope changes of a representative channel that exhibited LTP (Ch. 14). (**c**) Time course of mean fEPSP slopes of all 6 activated channels in the slice shown in (**a**). (**d-f**) *ASIC1a* KO mice. (**d**) The 64-channel overview of multisite synaptic responses recorded at baseline (*left*) and 3 hr after TBS (*right*) in an insular cortical slice prepared from an *ASIC1a* KO mouse. Asterisks indicate the stimulated channel (Ch. 29). Scale bar: 100 μV, 10 ms. (**e**) Time course of fEPSP slope changes of a representative channel that exhibited LTP (Ch. 21). (**f**) Time course of mean fEPSP slopes of all 10 activated channels in the slice shown in (**d**).

Supplementary Figure 8 by Li *et al.*

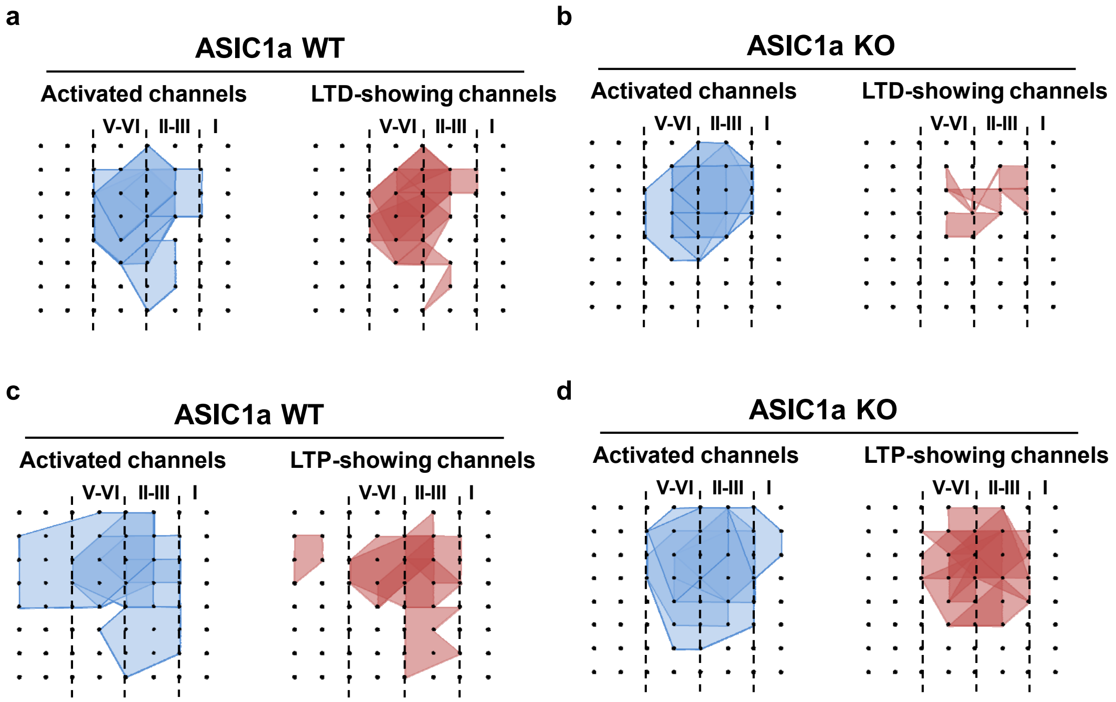

Supplementary Figure 8. Spatial network analysis of LTD (a, b) and LTP (c, d) induction in the insular cortical slices prepared from WT (a, c) and *ASIC1a* KO (b, d) mice.

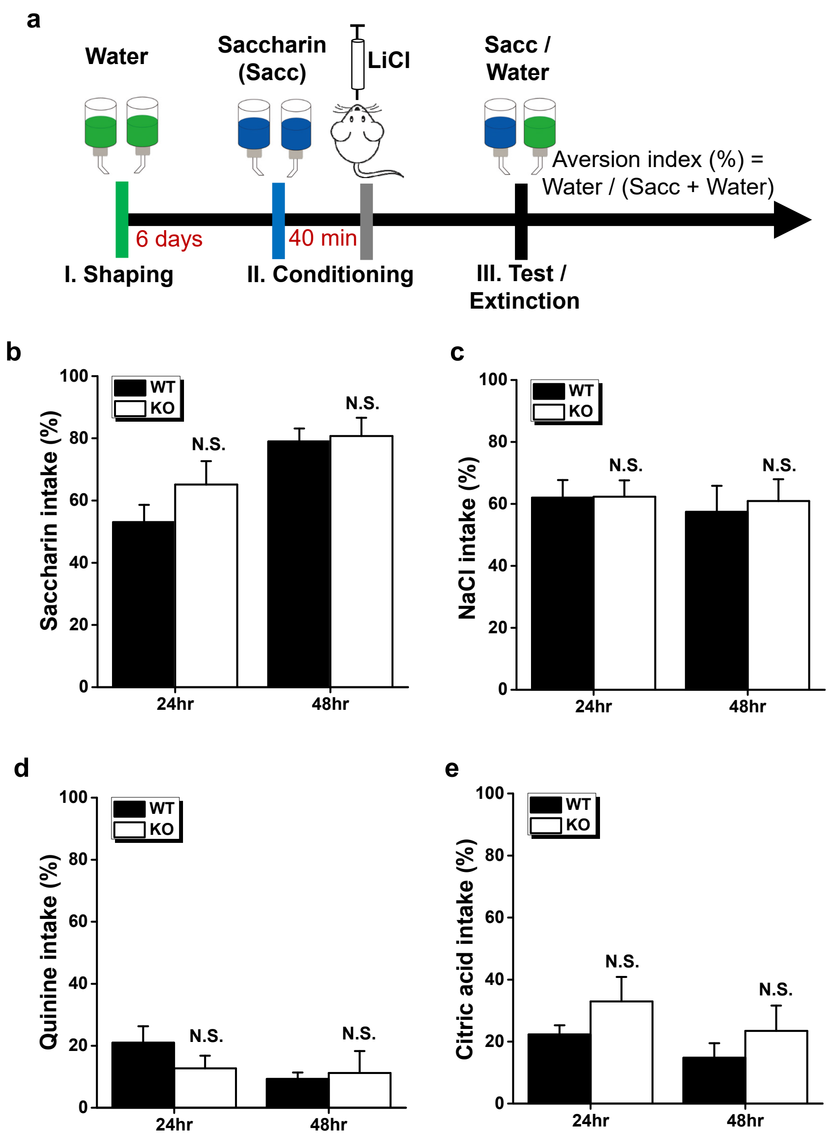

Supplementary Figure 9. Behavioral tests for taste preferences. (**a**) A general scheme of behavioral protocols used to establish and extinguish CTA. Please see text for details. (**b-e**) Unconditioned taste preference is not affected by the loss of ASIC1a. Intakes of four taste solutions (**b**, saccharin sodium, 0.5%, w/v; **c**, NaCl, 0.5%, w/v; **d**, quinine hydrochloride, 0.01%; **e**, citric acid, 10 mM) were compared against the intake of water (n = 7–13 per group) during two-bottle choice tests. N.S., not significant, P = 0.2033, 0.3810, 0.9721, 0.7556, 0.2289, 0.7951, 0.2067, and 0.3583 for the comparison of the 24 hr and 48 h taste preference for saccharin (b), NaCl (c), quinine (d), and acid (e), respectively, in WT *vs. ASIC1a* KO mice, unpaired Student's *t*-test.

Supplementary Figure 10 by Li *et al.*

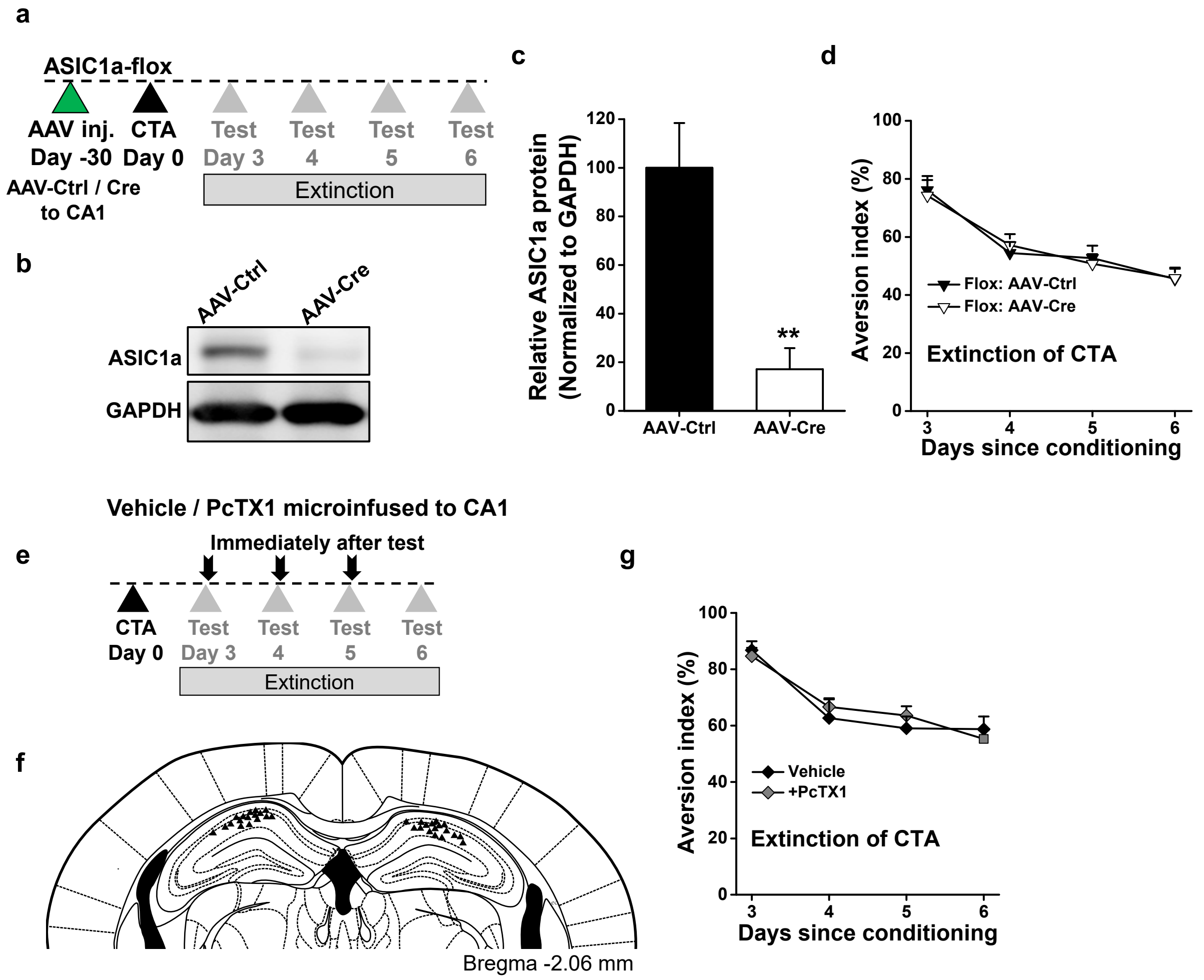

Supplementary Figure 10. ASIC1a in hippocampal CA1 plays a negligible role in CTA extinction. (**a**) Behavioral protocol used to test the effect on CTA extinction of region-specific knockout of ASIC1a from hippocampal CA1 neurons. *ASIC1a*$^{flox/flox}$ mice received bilateral injection of either AAV-Ctrl or AAV-Cre in the hippocampal CA1 region and were allowed to recover for approximately 30 days before CTA was established and two-bottle choice tests were performed for 4 consecutive days. (**b, c**) Representative immunoblots (**b**) and pooled data (**c**, n = 3 mice for each group) show that AAV-Cre reduced ASIC1a protein expression in hippocampal CA1 regions. $^{***}P <$ 0.01, P = 0.0076, AAV-Ctrl v*s.* AAV-Cre, unpaired Student's *t*-test. (**d**) Time courses of aversion indices over the 4-day period of CTA extinction for *ASIC1a*$^{flox/flox}$ mice that received injection of AAV-Ctrl or AAV-Cre in the hippocampal CA1 regions (n = 8 per group). Group, $F_{(1,64)} = 0.006$, $P = 0.939$; test day, $F_{(3,64)} = 19.004$, $P < 0.001$; interaction, $F_{(3,64)} = 0.142$, $P = 0.934$, two-way ANOVA. P = 0.7972, 0.6268, 0.7215, and 0.9544, for the comparison of aversion index on the 3, 4, 5, and 6 days since conditioning, receptively, in Flox: AAV-Ctrl *vs.* AAV-Cre groups, unpaired Student's

t-test. (**e-g**) Acute inhibition of ASIC1a channel in the hippocampal CA1 did not affect CTA extinction. (**e**) Behavioral protocol used to test the effect on CTA extinction of pharmacological inhibition of ASIC1a in hippocampal CA1 regions. Following the acquisition of CTA, WT mice received bilateral microinfusion of PcTX1 (10 μM, 0.5 μl) or vehicle immediately after the two-bottle choice test on days indicated. (**f**) Schematic of the infusion sites of hippocampal CA1 at the section of Bregma –2.06 mm. (**g**) Time courses of aversion indices over the 4-day period of CTA extinction for mice infused with PcTX1 or vehicle control into the hippocampal CA1 regions (n = 8–9 per group). Group, $F_{(1,68)} = 0.064$, $P = 0.806$; test day, $F_{(3,68)} = 18.778$, $P < 0.001$; interaction, $F_{(3,68)} = 0.488$, $P = 0.692$, two-way ANOVA. $P = 0.6308$, 0.6122, 0.4166, and 0.5467, for the comparison of aversion index on the 3, 4, 5, and 6 days since conditioning, receptively, in vehicle *vs.* PcTX1 groups, unpaired Student's *t*-test.

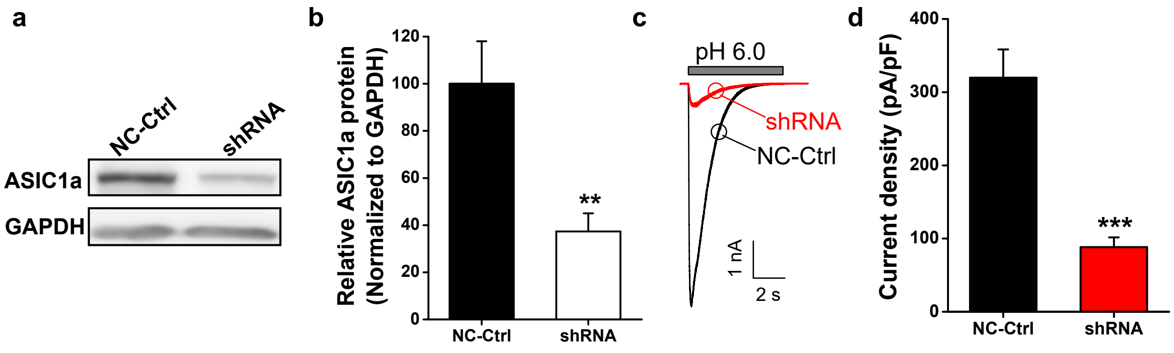

Supplementary Figure 11. *In vitro* analysis of the efficacy of shRNA against mouse ASIC1a. (**a**, **b**) cDNA construct of mouse ASIC1a was co-transfected with the negative control shRNA vector (NC-Ctrl) or ASIC1a-shRNA (shRNA) into CHO cells. After 48 hr, cell lysates were processed for Western blot analysis for ASIC1a and then re-blotted for GAPDH. Shown are representative images of Western blots (**a**) and quantification (**b**) of optical density of ASIC1a proteins normalized to that of GAPDH (n = 4 in each group). $^{**}P < 0.01$, $P = 0.0094$, NC-Ctrl *vs.* shRNA, unpaired Student's *t*-test. (**c, d**) Electrophysiological recordings of acid (pH 6.0)-induced currents in CHO cells that co-expressed mouse ASIC1a with NC-Ctrl or shRNA. Shown are representative current traces (**c**) and summary of peak current density evoked by the pH 6.0 solution (n = 10–12 cells in each group, **d**). $^{***}P < 0.001$, $P = 4.160\text{E-}05$, NC-Ctrl *vs.* shRNA, unpaired Student's *t*-test.

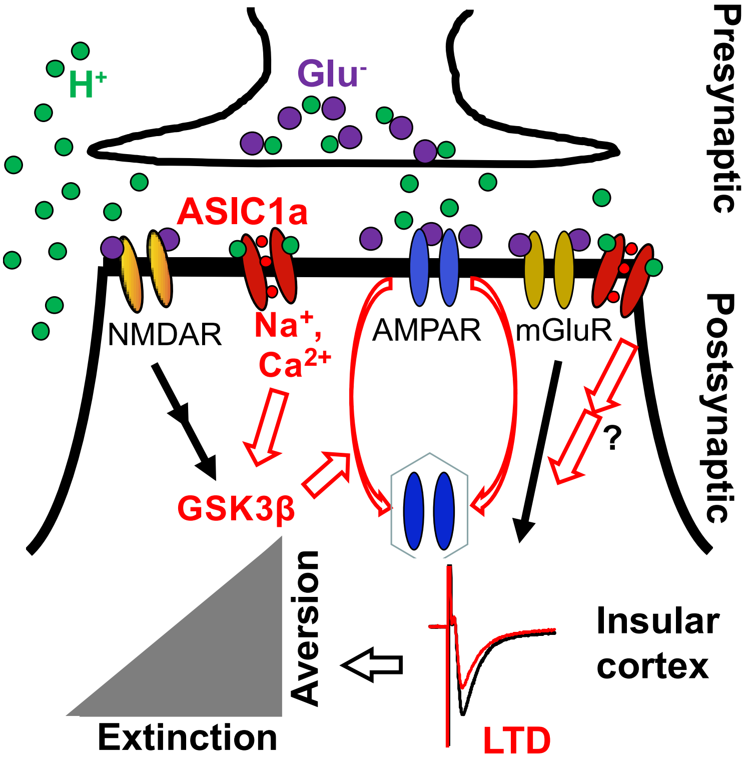

Supplementary Figure 12. A proposed scheme for ASIC1a regulation of insular LTD and its involvement in CTA extinction. Activation of ASIC1a leads to a rise in postsynaptic Ca^{2+} concentration, which in turn activates GSK3β (at least for NMDAR-dependent LTD). This triggers a series of downstream signaling cascades including AMPAR endocytosis and eventually results in the reduction of insular synaptic efficacy. The molecular mechanisms that underlie ASIC1a-dependent mGluR-mediated DHPG-LTD remain to be established. Behaviorally, the ASIC1a-dependent synaptic plasticity at insular synapses is critical for extinction of CTA. Please see text for more details.

Supplementary Figure 13 by Li *et al.*

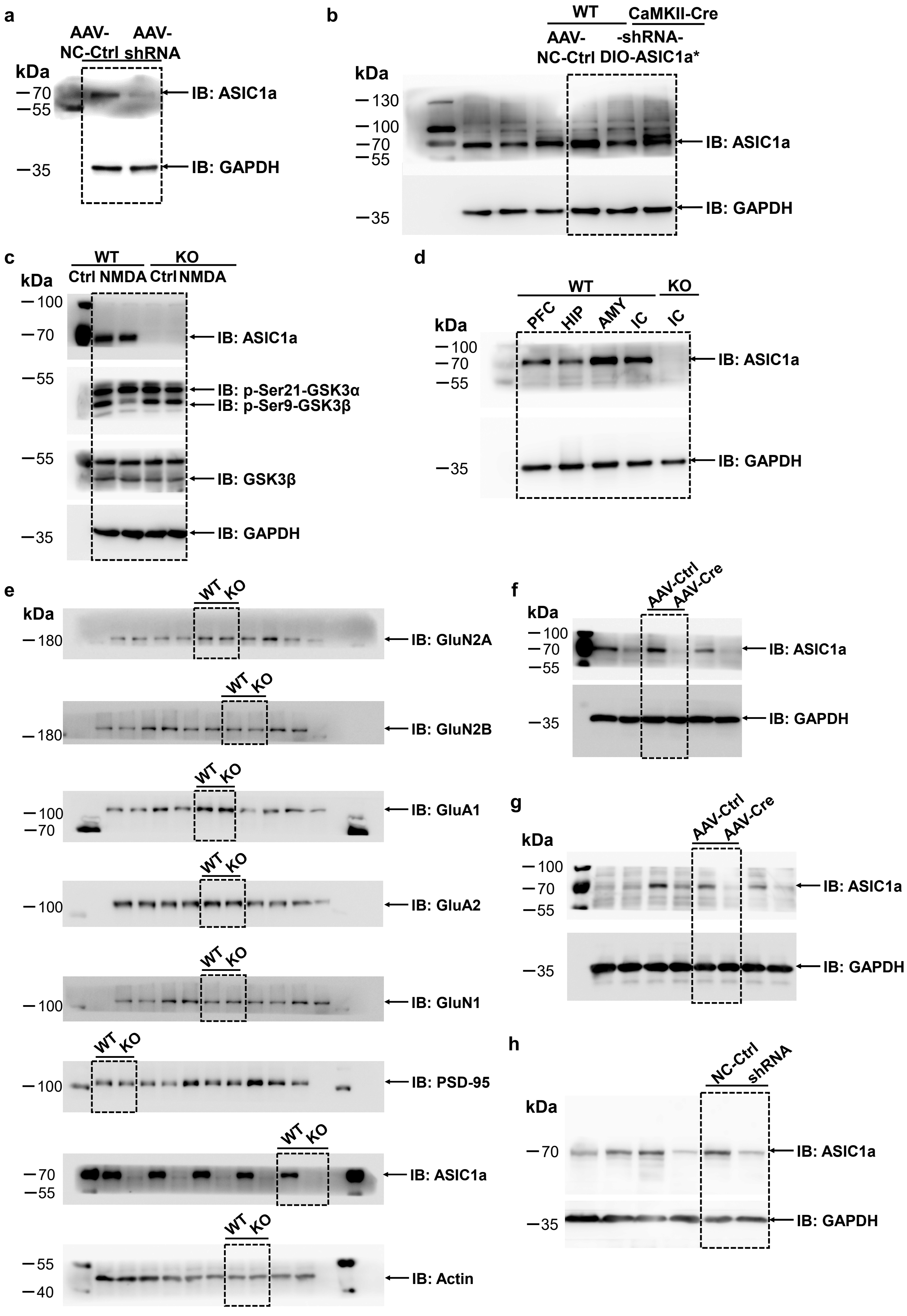

Supplementary Figure 13 Uncropped images of Western blots shown in *Figs. 5j* (a), *5o* (b), *6a* (c), and *Supplementary Figs. 1c* (d), *1d* (e), *5a* (f), *10b* (g), and *11a* (h).

Supplementary methods

Unconditioned taste preferences. The unconditioned taste preferences were tested according to a previous report[1] with modifications. Briefly, the ability of mice to discriminate between simple tastes was examined using the two-bottle, 2-day intake tests. Mice were housed in cages in which they had *ad libitum* access to food, but restricted (from 9:00 to 9:30 a.m.) access to water presented in one of the two bottles. During the one-week training, the other bottle was kept empty and the positions of the bottles were switched every 24 hr to train the mice to drink from either position. After training, the mice were given two bottles: one containing one of the four taste solutions [0.5% (w/v) sodium saccharin, 75 mM NaCl, 0.01% (w/v) quinine hydrochloride, or 10 mM citric acid], and the other containing only water. Intake from each bottle was recorded on the first day and again on the second day when the bottle positions were switched. Each mouse was tested for one taste solution. The preference index for the particular taste was calculated as follows: [taste solution intake (in grams)] / [taste solution intake (in grams) + water intake (in grams)] × 100% (Supplementary Fig. 9b-e).

Cell culture and electrophysiological recordings. The cDNA of mouse ASIC1a (GenBank accession: NM_009597.1) was expressed in Chinese hamster ovary (CHO) cells by transient transfection as reported previously[2]. In brief, CHO cells were cultured in F-12 Nutrient Mixture supplemented with 10% (v/v) fetal bovine serum and 1% (v/v) gluta-MAX^{TM-1} (Thermo Fisher Scientific) at 37°C in a humidified atmosphere of 5% (v/v) CO_2 and 95% O_2 (v/v) and passaged twice a week. Transient transfection of CHO cells was performed using HilyMax liposome transfection reagent (Dojindo Laboratories). The plasmid used contained, in addition to the desired ASIC1a cDNA, the coding sequence for enhanced green fluorescent protein (EGFP) to aid identification of transfected cells. Electrophysiological measurements were performed 24–48 hr after transfection.

Whole-cell patch-clamp electrophysiological experiments in cultured cells were conducted using the Molecular Devices (Foster City, CA) system (Axoclamp 200B, Digidata 1440, pClamp 10). In most experiments, 70–90% of the series resistance was compensated. Voltage-clamp (with the holding potential of –60 mV) recordings were performed as described previously[2]. Patch pipettes (3–5 MΩ) were filled with the following (in mM): 120 KCl, 30 NaCl, 10 HEPES, 5 EGTA, 2 MgATP, 1 $MgCl_2$ and 0.5 $CaCl_2$, adjusted to pH 7.2 with Tris-base. The incubation and recording solution contained (in mM): 150 NaCl, 5 KCl, 10 glucose, 2 $CaCl_2$, and 1 $MgCl_2$, buffered to pH 7.4 with either 10 HEPES, or to pH 6.0 with 10 2-(N-morpholino)-ethanesulfonic acid (MES). The osmolarity of solutions was maintained at 280–300 mOsm. The pH 6.0 solution was rapidly applied using the "Y-tube" method[3]. This system allows a complete exchange of the external solution surrounding a cell within 20 ms. All recordings were performed at room temperature (23 ± 2 °C).

Preparation of multi-electrode probe. A commercial 64-channel multisite recording system (MED64; Alpha-Med Sciences) was used for extracellular field potential recordings[4, 5]. The device (MED64 probe, P515A) had an array of 64 planar microelectrodes, each 50 × 50 μm in size, arranged in an 8 × 8 pattern (inter-electrode distance, 150 μm; Fig. 1a). Before use, the surface of the MED64 probe was treated with 0.1% polyethyleneimine (Sigma-Aldrich) in a 25 mM borate buffer (25 mM $Na_2B_4O_7$-$10H_2O$, adjust pH to 8.4 with HCl) overnight at room temperature. This coating helped to establish sufficient adhesion of the slice to the probe surface, enabling both reliable stimulation and a higher signal-to-noise ratio during recording. The probe surface was routinely rinsed three to five times with sterile distilled water immediately before use in each experiment. In general, the MED64 probes could be re-used for approximately 20–30 recording sessions with a mean duration of 4–6 hr. The electrode properties of the MED64 probes could be kept constant by carefully cleaning the probe with deionized water following each recording session.

Preparation of PSD fractions. The purification of the PSD fraction was performed based on previous studies[6, 7] with some modifications. Briefly, insular cortical tissue combined from 2 mice was homogenized in a buffer containing 5 mM HEPES (pH 7.4), 1 mM $MgCl_2$ and 0.5 mM $CaCl_2$ in the presence of protease inhibitors. The homogenized tissue was centrifuged at 1400 g for 10 min at 4 °C. The resulting supernatant (S1) was saved and the pellet was resuspended and centrifuged at 700 g resulting the supernatant (S1'). S1' was added to S1 and centrifuged at 13,800 g for 10 min to obtain a crude membrane fraction (P2 fraction). The P2 fraction was resuspended in 0.32 M sucrose and loaded onto a discontinuous sucrose gradient (from top, 0.85 M : 1.0 M : 1.2 M = 3 ml : 3 ml : 3 ml), and then centrifuged for 2 hr at 82,500 g in the SW-41 rotor (Beckman Coulter). The synaptosome fraction between 1 M and 1.2 M sucrose was collected with a syringe needle and resuspended in a buffer containing 6 mM Tris (pH 8.1) and 0.5% Triton X-100. After 15 min treatment by Triton X-100, the suspension was centrifuged at 201,800 g (Beckman Coulter) for 1 hr and the final pellet (PSD) was dissolved using a buffer containing 0.2% SDS and protease inhibitors. The "One-Triton" PSD was used because of the limited amount of the starting material.

Real-time reverse transcription polymerase chain reaction. Insular cortices of WT and *ASIC1a* KO mice were dissected and total RNA was extracted using TRIzol reagent (Thermo Fisher Scientific). Four μg of total RNA were used as a template for cDNA synthesis and amplification with the SuperScript III First-Stand Synthesis System (Thermo Fisher Scientific) according to the manufacturer's instructions. The cDNA was diluted to an equal concentration of 100 ng/μl and 100 ng of which was used for further PCR amplification. Real-time PCR was processed by the SYBR Premix Ex Taq^{TM} kit (Takara) using ABI PRISM 7000 Sequence Detection System (Applied Biosystems) with the following amplification conditions: 95 °C for 5 min;

40 cycles of 95 °C for 15 sec, 60 °C for 15 sec and 72 °C for 31 sec. The sequences of primers used in this study are listed as follows: *ASIC1a (NM_009597.1)* forward, 5'-CACCTTCCCTGCCGTCACTC-3', *ASIC1a* reverse, 5'-GCCCTGCTC TGTCGTAGAACTCA-3'; *ASIC1b (NM_001289791.1)* forward, 5'-GGCGAGCCCTTTAATCTCCA-3', *ASIC1b* reverse, 5'-ACTTCCCATACCGCGTGAAG-3'; *ASIC2a (NM_001034013.2)* forward, 5'-AGGGAGCCATGATGAGAACAT-3', *ASIC2a* reverse, 5'-GGTGTCAGCAGGCAATCTCC-3'; *ASIC2b (NM_007384.3)* forward, 5'-TGCCTTCATGGACCGTCTG-3', reverse, 5'- TGCCATCCTCGCCTGAGTTA-3'; *ASIC3 (NM_183000.2)* forward, 5'- CAGCCCTGTGGACCTGAG AA-3', *ASIC3* reverse, 5'- CGCCCTTAGGAGTGGTGAGC-3'; *ASIC4 (NM_183022.3)* forward, 5'-GCAGGAGGAATACCTACCCAT-3', *ASIC4* reverse: 5'-GCAGATAAGTTAGCCGCTGTTC-3'; *GAPDH (NM_001289726.1)* forward, 5'-AGTCAAGGCCGAGAATGGGAAG-3', *GAPDH* reverse, 5'-AAGCAGTTGGTGGTGCAGGATG-3'. To determine gene expression in insular cortices, threshold cycles for each transcript (Ct) were normalized to *GAPDH* (ΔCt). Calibrations and normalizations were performed using the $2^{-\Delta Ct}$ method in which *GAPDH* was used as the reference gene. Real-time PCR measurements were performed in triplicates.

SUPPLEMENTAL REFERENCES

1. Yu, H.*, et al.* Variant BDNF Val66Met polymorphism affects extinction of conditioned aversive memory. *J. Neurosci.* **29**, 4056-4064 (2009).
2. Yu, Y.*, et al.* A nonproton ligand sensor in the acid-sensing ion channel. *Neuron* **68**, 61-72 (2010).
3. Li, Y.F., Wu, L.J., Li, Y., Xu, L. & Xu, T.L. Mechanisms of H+ modulation of glycinergic response in rat sacral dorsal commissural neurons. *J. Physiol.* **552**, 73-87 (2003).
4. Liu, M.G.*, et al.* Long-term potentiation of synaptic transmission in the adult mouse insular cortex: multielectrode array recordings. *J. Neurophysiol.* **110**, 505-521 (2013).
5. Liu, M.G.*, et al.* Long-term depression of synaptic transmission in the adult mouse insular cortex in vitro. *Eur. J. Neurosci.* **38**, 3128-3145 (2013).
6. Carlin, R.K., Grab, D.J., Cohen, R.S. & Siekevitz, P. Isolation and characterization of postsynaptic densities from various brain regions: enrichment of different types of postsynaptic densities. *J. Cell Biol.* **86**, 831-845 (1980).
7. Jordan, B.A.*, et al.* Identification and verification of novel rodent postsynaptic density proteins. *Mol. Cell Proteomics* **3**, 857-871 (2004).
